# Assessing the individual and combined effects of ZnO nanoparticles and glyphosate on the gut microbiota of tadpoles

**DOI:** 10.3389/fmicb.2026.1751143

**Published:** 2026-03-26

**Authors:** Han Xue-dong, Guo Peng, Tong Qing, Yang Hong-sheng, Liu De-cai

**Affiliations:** College of Biology and Agriculture, Jiamusi University, Jiamusi, China

**Keywords:** amphibian health, ecotoxicology, glyphosate exposure, gut microbiota, microbial diversity, zinc oxide nanoparticles

## Abstract

**Introduction:**

Freshwater co-contamination by zinc oxide nanoparticles (ZnO NPs) and glyphosate poses a rising threat to amphibians. While the gut microbiota is central to host metabolism and immunity, the individual and synergistic impacts of these pollutants on tadpole microbial homeostasis—and the resulting vulnerability—remain largely unexplored.

**Methods:**

This study examines the impact of ZnO NPs and glyphosate, alone and combined, on the gut microbiota of *Rana dybowskii* tadpoles. Treatments included exposure to ZnO NPs, glyphosate, both, or a control. Total DNA was extracted from intestinal contents samples, and the V3-V4 region of the bacterial 16S rRNA gene was amplified and sequenced on an Illumina MiSeq platform.

**Results:**

Diversity analyses revealed significant alterations in alpha and beta diversity indices, with the ZnO and glyphosate treatments individually and synergistically disrupting microbial communities. The gut microbiota dysbiosis index (MDI) was elevated in treated groups, indicating microbial imbalance. Dominant phyla such as Firmicutes, Proteobacteria, and Actinobacteriota exhibited significant shifts in abundance across treatments. Functional pathway predictions via Kyoto Encyclopedia of Genes and Genomes (KEGG) analysis highlighted disruptions in metabolic processes, immune function, and disease-related pathways. Ecological assembly models suggested that deterministic processes predominantly governed microbiota changes under pollutant exposure. These findings underscore the detrimental impact of ZnO NPs and glyphosate on amphibian gut microbiota, with potential implications for amphibian health and ecosystem stability.

**Discussion:**

By integrating diversity metrics, a dysbiosis index, and community assembly models, we show that ZnO NPs and glyphosate can shift the tadpole gut microbiota toward an alternative functional state. This links mixture pollution to microbiota-mediated vulnerability and provides microbial biomarkers and mechanistic targets for amphibian risk assessment.

## 1 Introduction

Amphibians are presently confronting a critical extinction catastrophe, as numerous species undergo significant population declines attributable to habitat destruction, climate change, pollution, illnesses, and invasive species, leading to an escalating extinction rate (Wake and Vredenburg, 2008; Long et al., 2024; Annamalai et al., 2024). In this context, the health of the gut microbiota is particularly crucial, as it plays an important role in maintaining the physiological balance of amphibians and enhancing their ability to withstand environmental stress (Long et al., 2024; Tong et al., 2020; Yang et al., 2024; Han et al., 2025). Global nanotechnology is developing rapidly, with a wide range of nanoparticles and products, and their safety has attracted much attention, the widespread use of herbicides such as glyphosate also poses potential risks (Knutie et al., 2018; Wu et al., 2023). These environmental pollutants not only pose a direct threat to the survival of amphibians, but also disturb the balance of their gut microbiota, thereby further impairing their physiological functions and resilience to stress (Han et al., 2025). Although amphibian gut microbiota research has progressed, evidence remains limited regarding nanoparticle-associated shifts in amphibian gut bacterial community composition and inferred functional potential, particularly under co-exposure with pesticides (Yang et al., 2024).

Zinc oxide nanoparticles (ZnO NPs) may pose a serious threat to the gut microbiota of aquatic organisms (Wu et al., 2023). Besides traditional pollutants like heavy metals, pesticides, and chemicals, emerging pollutants such as microplastics and nanomaterials are also growing concerns (Chen et al., 2022; Chen et al., 2022). The global annual production of ZnO NPs reaches 100,000 tons, and they enter aquatic environments through agricultural runoff, industrial wastewater, consumer product emissions, atmospheric deposition, and incomplete sewage treatment (Chen et al., 2022; Gomes et al., 2023; Murthy et al., 2022). This widespread presence raises concerns about the environmental impact of ZnO NPs (Wu et al., 2023). Recent research has found that they pose risks in aquatic environments, inhibiting animal growth, increasing mortality, accumulating in tissues, and causing oxidative stress and gene function inhibition (Wu et al., 2023; Chen et al., 2022; Long et al., 2023). ZnO NPs affect the gut microbiota directly and indirectly through various mechanisms, such as oxidative stress, antimicrobial activity, physical damage, and metal ion release, all of which contribute to microbiota dysbiosis (Gomes et al., 2023; Murthy et al., 2022). For instance, in the study of *Carassius auratus*, exposure to ZnO NPs for 14 d induced marked alterations in the diversity and structure of the gut microbiota, further emphasizing their disruptive impact on microbial balance (Wu et al., 2023). Considering that early microbial colonization in amphibian larvae is critical for gut development and immune system maturation, it is essential to determine whether ZnO NPs disrupt the succession of the gut microbiota during this period (Wu et al., 2023; Chen et al., 2022; Long et al., 2024). However, the research on the mechanisms of ZnO NPs’ impact on amphibian gut microbiota remains in its infancy and requires further investigation.

The widespread use of glyphosate may have a significant impact on the gut microbiota and health of amphibians (Knutie et al., 2018). Glyphosate, a widely used herbicide, has an annual production exceeding 1 million tons (Benbrook, 2016; Klingelhöfer et al., 2021). Glyphosate enters aquatic environments via spray drift, leaching, and runoff, contaminating surface waters and threatening aquatic ecosystems, including amphibians (Borggaard and Gimsing, 2008). Glyphosate not only inhibits the growth of aquatic animals but also affects their early development, behavior, and physiological health (Lopes and Moraes, 2022; Sulukan et al., 2023). Due to the indicative role of amphibians in ecosystems, their sensitivity to glyphosate highlights the potential environmental hazards of this chemical (Knutie et al., 2018; Edge et al., 2013). For example, tadpoles are highly sensitive to glyphosate, with a 96-h LC_50_ of approximately 1.0–10 mg/L (Mann and Bidwell, 1999; Wagner et al., 2013). Recently, the influence of glyphosate on animal gut microbiota has emerged as a study focus, encompassing insects like bees, fish, mammals such as mice and pigs, and crustaceans including the Chinese mitten crab (Motta et al., 2018; Yan et al., 2023; Rani et al., 2023; Tang et al., 2020; Yang et al., 2019). These studies typically use metagenomics and metabolomics techniques to explore how glyphosate affects gut microbial balance and its impact on host physiology (Yan et al., 2023). In addition, some studies have been conducted on amphibians (Boccioni et al., 2021; Villatoro-Castañeda et al., 2023). For example, exposure to Roundup (glyphosate) and antibiotics altered the gut microbiota of *Rana berlandieri* tadpoles, affecting their growth and behavior (Villatoro-Castañeda et al., 2023). Glyphosate and ciprofloxacin exposure had a negative impact on the gut microbiota of *Rhinella arenarum* tadpoles (Boccioni et al., 2021). Although these studies provide preliminary insights into the effects of glyphosate on amphibian gut microbiota, related research remains relatively limited and requires further investigation.

Joint toxicity research, especially concerning the combined effects of pollutants like ZnO NPs and glyphosate, has become a critical focus in environmental science due to its impact on ecosystems, organisms, and gut microbiota (Han et al., 2025; Chen et al., 2022; Sun et al., 2022). Current studies aim to elucidate pollutant interaction mechanisms, the toxic effects of pollutant mixtures, their impact on ecosystems, and the development of new methodologies (Zhang et al., 2022; Wu et al., 2016). Combined pollutant exposure can induce toxic effects via mechanisms like oxidative stress, gene expression regulation, immune interference, and gut microbiota disruption (Yan et al., 2020; Wang et al., 2022). Pollutant mixtures, such as heavy metals with pesticides or microplastics with chemicals, significantly alter aquatic biodiversity, disrupt ecosystem functions, and modify gut microbiota composition (Chen et al., 2022; Burgos-Aceves et al., 2024). In joint toxicity research, the gut microbiota is crucial for organismal health (Han et al., 2025). In the field of joint toxicity research, the gut microbiota plays a crucial role as an indicator and participant in organismal health (Burgos-Aceves et al., 2024; Liu et al., 2022). While the mechanisms through which pollutants affect the microbiota remain underexplored, various pollutant combinations have been shown to significantly alter the gut microbiota of different species (Boccioni et al., 2021; Gonçalves and de Almeida, 2023). For example, the impact of cadmium and triadimefon have significant negative effects on *R. dybowskii* survival, body mass and gut and skin microbiota (Han et al., 2025; Zhu et al., 2025). Exposure of *Cyprinus carpio* to polyethylene microplastics and glyphosate alters the abundance and diversity of its gut microbiota (Chen et al., 2022). Therefore, combined exposure can affect gut microbiota structure and function through mechanisms like immune responses, chemical toxicity, metabolic changes, microbial interactions, and long-term cumulative effects (Sun et al., 2022). Given the importance of pollutant impacts on gut microbiota and joint toxicity complexity, the scarce research on ZnO NPs and glyphosate interaction urgently needs in-depth study.

*Rana dybowskii* is mainly distributed in the mountainous streams and nearby forests of northeastern China (Long et al., 2024; Long et al., 2024). As an important intermediate link in the ecosystem, *R. dybowskii* is both a predator and prey, maintaining ecological balance (Tong et al., 2020). The habitat (northeastern China) of the *R. dybowskii* is also an important region for both agriculture and industry, widely uses glyphosate as a herbicide in agriculture, while industrial production releases a large amount of ZnO NPs (Li et al., 2024; Guo et al., 2022). Consequently, *R. dybowskii*, which may inhabit and reproduce in contaminated ponds, has a substantial risk of exposure to both nanoparticles and pesticides (Peluso et al., 2023; Murthy et al., 2023). The life cycle of *R. dybowskii* includes four stages: egg, tadpole, metamorphosis, and adult, with the tadpole and metamorphosis stages being the most sensitive to environmental stress and pollution (Tong et al., 2020; Long et al., 2024; Villatoro-Castañeda et al., 2023). The individuals are highly sensitive to water quality and the ecological environment, and the gut microbiota plays a crucial role in intestinal development and the maturation of the immune system during tadpole and metamorphosis stages (Long et al., 2024; Long et al., 2024). This study exposed *R. dybowskii* tadpoles to ZnO NPs, glyphosate, or both. Changes in gut microbiota composition and function were then examined using Illumina MiSeq sequencing of 16S rRNA gene amplicons, followed by analyses of diversity, microbial dysbiosis indices, and functional pathways. We hypothesize that exposure to ZnO NPs and glyphosate, individually and in combination, significantly disrupts the composition and diversity of the gut microbiota in *R. dybowskii* tadpoles, leading to increased microbial dysbiosis and compromised physiological health.

## 2 Materials and methods

### 2.1 ZnO NPs and chemical materials

Pristine ZnO powder (nominal primary particle size: 30 nm, as specified by the manufacturer) was purchased from Qinghe County Kangshuo Welding Materials Co., Ltd. (Xingtai, China) and used in the exposure experiments (Salla et al., 2023). The hydrodynamic diameter of ZnO NPs (10 mg/L) was measured by dynamic light scattering (DLS) on a Zetasizer Nano ZS90 (Malvern, UK) at room temperature (25 ± 2 °C). The morphology of ZnO NPs (10 mg/L) was characterized by transmission electron microscope (TEM, JEM-2100, JEOL, Japan). Lattice-resolved images were further acquired in high-resolution TEM (HRTEM) mode on the same instrument. Glyphosate (C_3_H_8_NO_5_P, exceeding 95% purity) was acquired from Wengjiang Reagent Co. Ltd. (Guangdong, China), and stock solutions of 10 mg/L were prepared (Chen et al., 2022; Cattaneo et al., 2011; Liu et al., 2021).

### 2.2 Experimental tadpoles

*Rana dybowskii* frogs were gathered from Heilongjiang Province, China’s Luobei County (47.5789 N, 130.3794 E). Frogs of both sexes were taken from a hibernating pond in November as they started to fast because of the dropping temperatures. The frogs were in perfect condition, weighing 20.48 ± 1.16 g. Over the winter, hibernation took place in wintering ponds at university laboratories. After mating and spawning in the lab at Jiamusi University, tadpoles were produced in March. Tadpoles were reared until Gosner stage 20 (G20; 0.12 ± 0.01 g). At this stage (G20), tadpoles were randomly assigned to treatments, and exposure was initiated. (Gosner, 1960). After 28 days of exposure, tadpoles reached Gosner stage 26 (G26) and were collected for subsequent analyses (Han et al., 2025; Long et al., 2024).

### 2.3 Experimental design

#### 2.3.1 Exposure experiment

In studies of *Oreochromis niloticus*, ZnO NPs at concentrations of 5–30 mg/L have been shown to cause significant toxicological and physiological effects (Kaya et al., 2015). Further research on *Carassius auratus* demonstrated that exposure to 1–5 mg/L ZnO NPs can induce notable disturbances in the intestinal microbiota as well as fluctuations in immune indicators in skin mucus, among other physiological changes, while concentrations of 25–50 mg/L can lead to more pronounced histological damage (Wu et al., 2023). Accordingly, based on documented concentrations for water environments and other toxicological research, 10 mg/L was selected as the exposure concentration for ZnO NPs. In toxicological studies examining glyphosate in fish, exposure at concentrations of 5, 10, 15, and 20 mg/L has been shown to alter the brain-gut axis, serum biochemistry, and oxidative stress indicators in *Cyprinus carpio L.* (Chen et al., 2022; Cattaneo et al., 2011; Liu et al., 2021). Hence, 10 mg/L was used as the glyphosate exposure concentration.

The experiment involved four groups: the C group (control, 0 mg/L), G group (glyphosate, 10 mg/L), Z group (ZnO NPs, 10 mg/L), and ZG group (glyphosate, 10 mg/L + ZnO NPs, 10 mg/L). Each group consisted of three replicates, with 35 tadpoles in each container. The container is a 10 L white polyethylene cylinder with a top outer diameter of 25 cm, a bottom outer diameter of 19 cm, and a height of 33 cm. An experiment was conducted over 28 d to assess the impact of toxic substances on tadpole gut microbiota.

During the experiment stage, tadpoles were maintained under consistent water quality conditions. A total of 35 tadpoles were present in a bucket containing 10 L of solution. The experimental setup consisted in a water temperature of 15.9 ± 1.8 °C and daily feeding of tadpoles from fish meal flakes and rabbit pellets, both supplied by Dolphin Aquarium Co., Ltd., Pengjiang District (Hernández-Gómez et al., 2020; Mirč et al., 2023). The lighting was configured for a 12-h day-night cycle. Solutions were refreshed every 3 d throughout the 28-day trial, with 50% of the tank water changed with dechlorinated water to reduce metabolic waste buildup, and ZnO NPs and glyphosate were reintroduced to sustain consistent levels (Wu et al., 2023; Hatami et al., 2019). Every bucket was daily cleaned using a long pipette to remove waste and dead tadpoles, therefore preserving water cleanliness.

#### 2.3.2 Sample collection

On day 28, tadpoles (G26) were promptly sent to the laboratory at Jiamusi University. The tadpoles were euthanized by direct immersion in a combination of tricaine methanesulfonate (MS-222, supplied by Shengyuan Aquatic Products Co., Ltd., Jinjiang, Fujian) and alcohol within a glass desiccator (Hernández-Gómez et al., 2020; Shu et al., 2022). Absence of responsiveness to stimuli and indicators of rigor mortis and tissue degradation proved mortality (Forgione and Brady, 2022). We collected gut samples after rinsing tadpoles with ultrapure water (Shu et al., 2022). Approximately nine samples were collected from Group C and assigned identifiers 1 through 9. Concurrently, six samples each were collected from Groups G, Z, and ZG, labeled 1 through 6, for 16S rRNA gene sequencing.

Following euthanasia, the gastrointestinal system was carefully split from the body, from the anal area up to - but not including - the stomach. By 10 min post-mortem, both the small and large intestines were removed. The intestinal contents were painstakingly extracted into a sterile 5 mL container. Every specimen was gathered using fresh sets of aseptic tweezers to avoid cross-contamination. Every gut sample was kept in a sterile container and frozen at −80 °C.

### 2.4 DNA extraction and PCR amplification

Following the recommended procedure, microbial DNA from the gut microbiota was extracted by homogenizing materials and using a FastDNA® spin kit for soil (MP Biomedical, US). A NanoDrop 2000 spectrophotometer (Thermo Scientific, US) was used to detect the DNA content and A260/A280 ratio, while 1% agarose gel electrophoresis was used to assess the quality of the DNA. With the use of the primers 338F (5′-ACTCCTACGGGAGGCAGCAG-3′) and 806R (5′-GGA CTACHVGGGTWTCTAAT-3′), the V3-V4 regions of the bacterial 16S rRNA genes were amplified. The PCR procedure included 3 min of denaturation at 95 °C, 27 cycles of denaturation at 95 °C for 30 s, annealing at 55 °C for 30 s, and extension at 72 °C for 45 s, and a final extension at 72 °C for 10 min. In order to reach a final volume of 20 μL, the PCR mixture contained 4 μL of 5 × FastPfu buffer, 0.4 μL of FastPfu polymerase, 2 μL of 2.5 mM dNTPs, 10 ng of template DNA, 0.8 μL of each 5 μM primer, and sterile ddH_2_O. The PCR findings were separated using a 2% agarose gel and then purified using Axygen Biosciences’ AxyPrep DNA gel kit. The DNA was quantified using a Promega QuantiFluor™-ST assay kit.

### 2.5 Illumina MiSeq

The samples were subjected to quality evaluation, quantification, and sequencing using paired-end 2 × 300 reads on a US Illumina MiSeq platform after the amplicon levels were standardized. Microbiota sequencing data generated in this study have been deposited in the NCBI SRA under BioProject accession numbers PRJNA1208430, PRJNA1208426, and PRJNA1050064. The correspondence between the BioProject accession numbers, experimental groups, and the associated NCBI BioSample accession numbers is provided in the Supplementary Materials (Supplementary Table S1).

### 2.6 Processing of sequencing data

The raw FASTQ files underwent meticulous processing through an intricate bioinformatics approach. Initially, sequences of 300 base pairs (bp) with an average Phred quality score below 20 were trimmed utilizing a sliding window of 50 bp, aiming to preserve sequences of 50 bp or longer post-trimming. Sequence assembly was restricted to reads with a minimum overlap of 10 bp in a consecutive manner, while misaligned reads were discarded. Stringent quality control measures were subsequently applied, removing sequences with erroneous barcodes, primer region mismatches exceeding one base pair, or ambiguous nucleotides. Following quality enhancement, operational taxonomic units (OTUs) were clustered using UPARSE version 7.1 at a 97% similarity threshold, and chimeras were eliminated via UCHIME. The remaining 16S rRNA sequences were classified by aligning them with the Silva (SSU138) 16S rRNA reference database, employing a rigorous confidence level of 70%.

### 2.7 Ecological and statistical analysis

This study used mothur v.1.30.2^1^ to generate rarefaction curves and evaluate gut microbiota alpha diversity metrics, including Chao, Shannon and observed species (Sobs). The examination of alpha diversity was performed using the Kruskal–Wallis H test, followed by post hoc pairwise comparisons. Only statistically significant *p* values (*p* < 0.05) were reported. The gut microbiota dysbiosis index (MDI) was analyzed using species-level taxonomic analysis with MetaPhlAn2, and graphics were generated in R (vegan 2.4.3) package (Gupta et al., 2020; Gunathilake et al., 2020). The software, packages, and versions used in this study are summarized in Supplementary Table S2.

Beta diversity distance matrices were calculated in QIIME^2^ using Bray–Curtis dissimilarity and weighted UniFrac distance based on the OTU data. Non-metric multidimensional scaling (NMDS) analysis and its graphical representation were generated in R (vegan 2.4.3), and differences among the C, Z, G, and ZG groups were tested using analysis of similarities (ANOSIM) and multivariate non-parametric variance analysis models (Adonis, with 999 permutations) (Lozupone and Knight, 2005; Caporaso et al., 2010).

To explore community assembly processes, Sloan et al. (2006) neutral community model (NCM) was used to relate OTU relative abundance to occurrence frequency. Deterministic and stochastic assembly processes were inferred using a null-model framework based on the *β*-nearest taxon index (βNTI) and the Raup–Crick metric based on Bray–Curtis dissimilarity (RCBray), implemented in the “picante” and “ecodist” packages (Stegen et al., 2013; Zhou et al., 2023). In addition, infer community assembly mechanisms by phylogenetic bin-based null model analysis (iCAMP) was applied as a phylogenetic bin-based approach to quantify the relative importance of assembly processes (Ning et al., 2020).

Shared and unique OTUs among groups were summarized using Venn diagrams (Shade and Handelsman, 2012). To classify dominant microbial community structures across the C, G, Z, and ZG groups, samples were clustered in R (v3.3.1) using Jensen–Shannon divergence (JSD) with the ade4, cluster, and clustersim packages to identify enterotypes.

The dominant species and their relative abundances in each sample were analyzed using R version 3.3.1 with data from the ‘tax_summary_a’ folder. To assess differences in relative abundance across groups, the Kruskal–Wallis H test was applied, followed by the Benjamini-Hochberg FDR correction for multiple comparisons, with a significance threshold set at *p* < 0.05. A ternary plot was created using the ggtern and ggplot2 packages to visualize the distribution and interactions of dominant species (those exceeding 0.5% in any sample) among the different groups (Xie et al., 2023). Linear discriminant analysis effect size (LEfSe) with an LDA > 4 was employed to identify specific phyla and genera, integrating both statistical significance and biological relevance in the process (Lian et al., 2019). The indicator taxa connected to each group were identified by the indicator value (IndVal) method (Dufrêne and Legendre, 1997).

Microbial phenotypes were predicted using BugBase and compared among groups using the Kruskal–Wallis test (Lucas et al., 2018). Differences were considered significant at *p* < 0.05. Phylogenetic Investigation of Communities by Reconstruction of Unobserved States 2 (PICRUSt2) was used to infer functional potential from 16S rRNA gene sequences by predicting Kyoto Encyclopedia of Genes and Genomes (KEGG) ortholog (KO) and pathway profiles (Kanehisa et al., 2012; Langille et al., 2013).

## 3 Results

### 3.1 Characterization of ZnO nanoparticles

As shown in Supplementary Figure S1, the particle size and polydispersity index (PDI) of ZnO NPs measured by DLS were 112.7 ± 2.2 nm and 0.034 ± 0.022, and the intensity distribution was unimodal (Peak 1 accounted for 100%). At low magnification, these nanoparticles appeared as irregular aggregates, which were composed of nanoscale particles (Supplementary Figures S2A,B). The high-magnification image (Supplementary Figures S2C,D) showed that the particle outline was clear and exhibited a polyhedral morphology. Continuous lattice fringes can be observed in the high-resolution image (Supplementary Figures S2E,F).

### 3.2 Gut microbiota dysbiosis index, alpha diversity and shared microbiota

Illumina MiSeq sequencing produced 2,332,005 high-quality sequences from 27 samples (mean 86,371 reads per sample; median 82,999), grouped into 894 OTUs, with 414 bp/read. Rarefaction and Shannon curves (Supplementary Figure S3) validated sufficient sequencing depth, the former’s plateau and the latter’s flattening showing adequacy for gut microbiota analysis.

**Figure 1:**
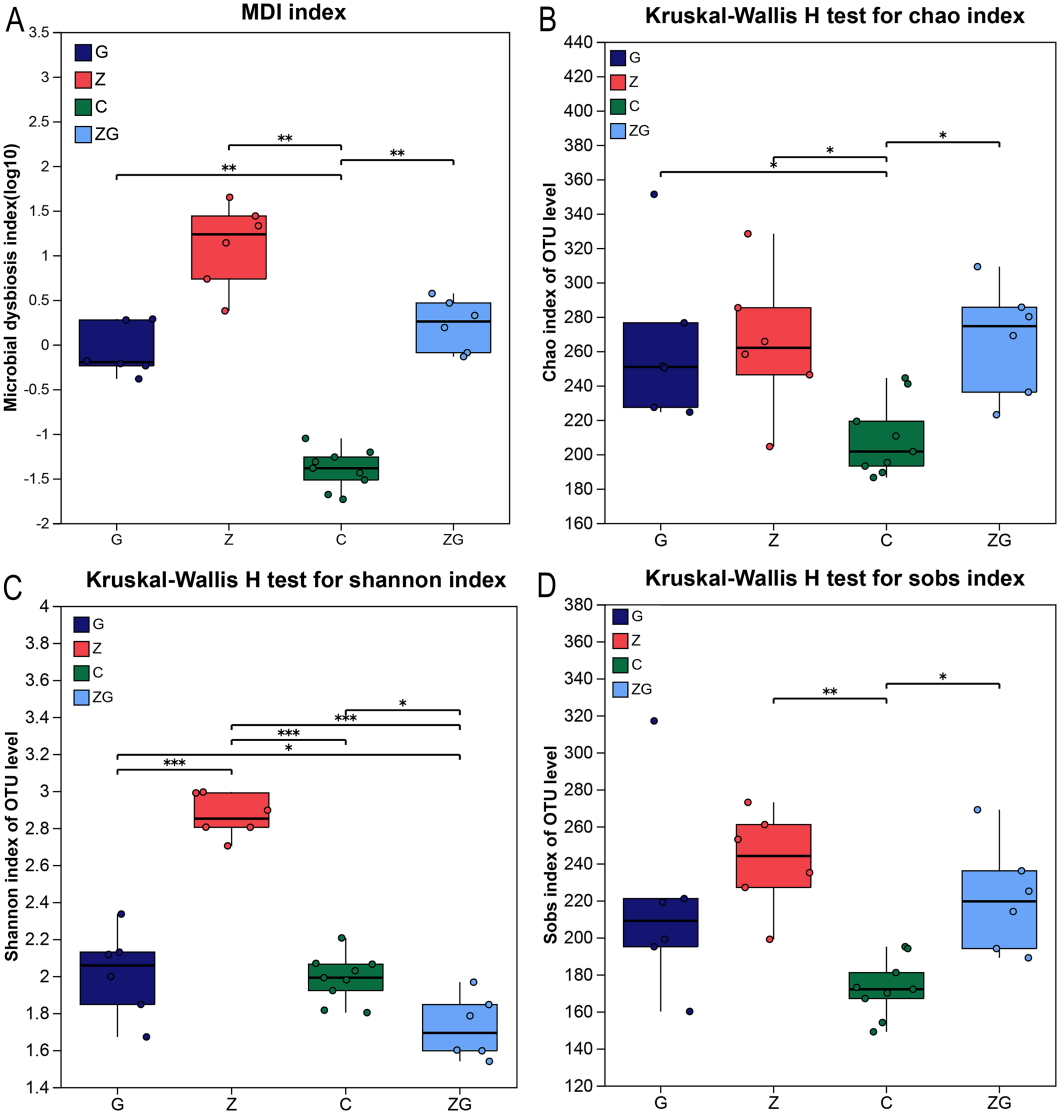
Comparison of microbial dysbiosis index (MDI) and alpha diversity across different experimental groups. **(A)** The distribution of the MDI across C, G, Z, and ZG to assess the effects of environmental pollutants on gut microbial dysbiosis. The line inside the box indicates the median value, reflecting the central tendency of the MDI in each group. **(B)** The Shannon index distribution for the C, G, Z, and ZG experimental groups reflects the gut microbiota diversity, with higher values indicating greater diversity. **(C,D)** Show the distributions of the Chao and Sobs indices, reflecting estimated and observed species richness, respectively (higher values indicate greater richness).

The gut microbiota dysbiosis of each group showed significant differences, as revealed by the MDI index. The C group exhibited lower indices of microbial dysbiosis compared to the other three groups (Figure 1A). The MDI indices of the ZG groups were higher than those of the C group but lower than those of the Z groups (Wilcoxon rank sum test, multiple test correction: FDR, two-sided test, *p* < 0.05; Figure 1A). Significantly differences were noted in Chao, Shannon, and Sobs indices among C, G, Z, and ZG groups (Kruskal–Wallis H test, *p* < 0.05; Figures 1B–D).

The Shannon indexes showed that there were significant differences in the gut microbial diversity among the groups (one-way ANOVA, Tukey–Kramer, *p* < 0.05; Figure 1B). Group C had a total of 357 OTUs, Group G had 470 OTUs, Group Z had 505 OTUs, and Group ZG had 473 OTUs. With a shared subset of 141 OTUs across four experimental groups (Supplementary Figure S4C). The C group had 95 unique OTUs, the G group 108, the Z group 139, and the ZG group 72 (Supplementary Figure S4C). There were 26 shared OTUs in groups C and G, 19 shared OTUs in groups C and Z, 14 shared OTUs in groups C and ZG, 29 shared OTUs in groups G and Z, 68 shared OTUs in groups Z and ZG, and 34 shared OTUs in groups G and ZG (Supplementary Figure S4C).

### 3.3 Beta diversity, ecological assembly of gut microbiota by NCM

**Figure 2:**
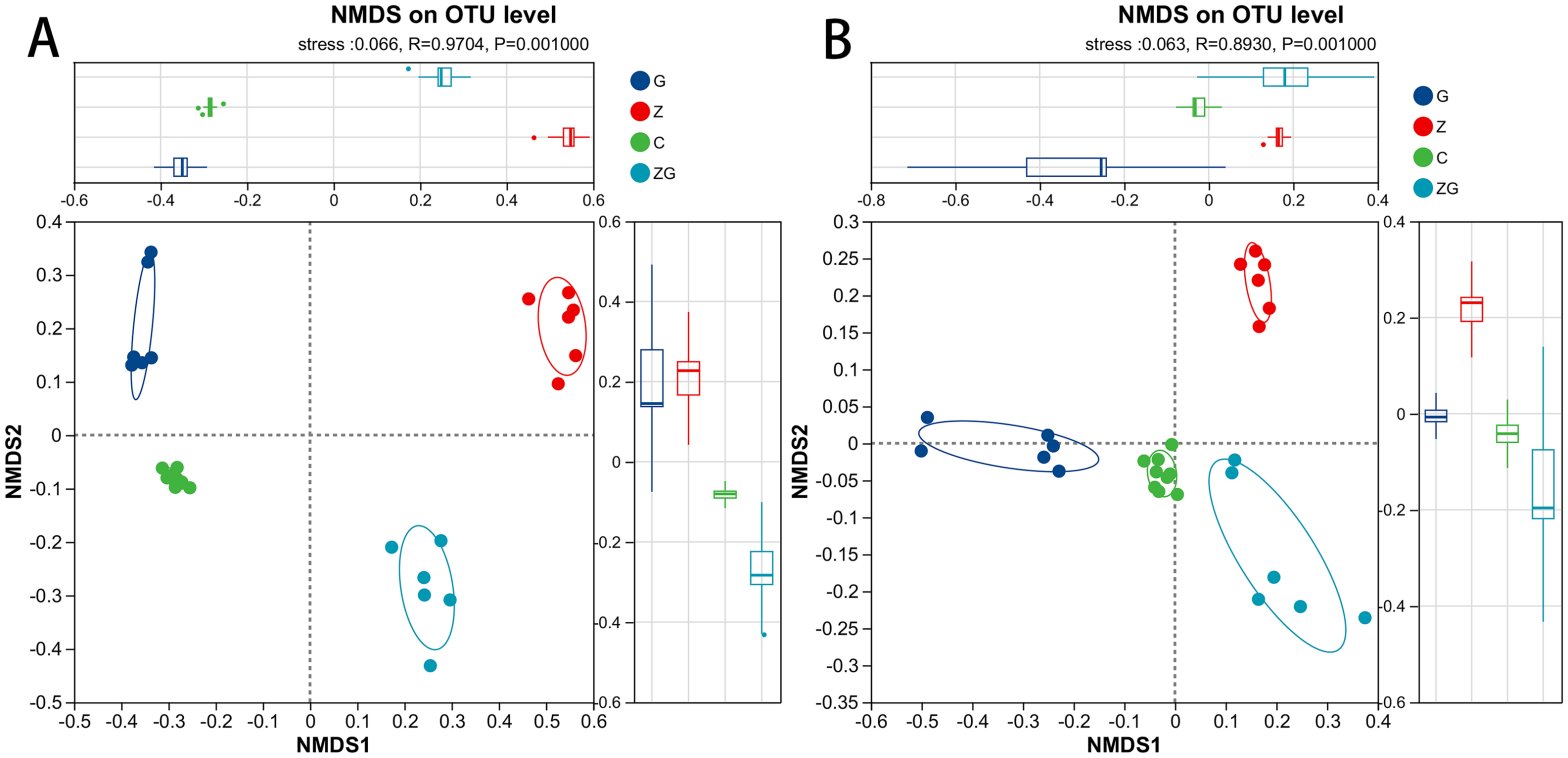
Non-metric multidimensional scaling (NMDS) analysis of gut microbiota differences among the all groups in *R. dybowskii.* To compare the effects of C vs. G vs. Z vs. ZG on gut microbiota community clustering and dispersion, Bray–Curtis dissimilarity **(A)** (stress = 0.066) and weighted UniFrac distances **(B)** (stress = 0.063) were calculated using an OTU-level. Each data point represents an individual sample from the gut system, and the proximity of sample points reflects their similarity. The closer the points, the greater the similarity between samples.

The gut microbiota of C, G, Z, and ZG groups differed significantly, shown by Bray–Curtis (Adonis: *R*^2^ = 0.824, *p* = 0.001; ANOSIM, 0.970, *p* = 0.001; Figure 2A) and weighted UniFrac (Adonis: *R*^2^ = 0.773, *p* = 0.001; ANOSIM, 0.893, *p* = 0.001; Figure 2B). The C group was positioned among the other three groups, and was relatively close to the G group (Figure 2). The G group was close to the ZG group. Compared to the other groups, the Z group was farther away (Figure 2).

**Figure 3:**
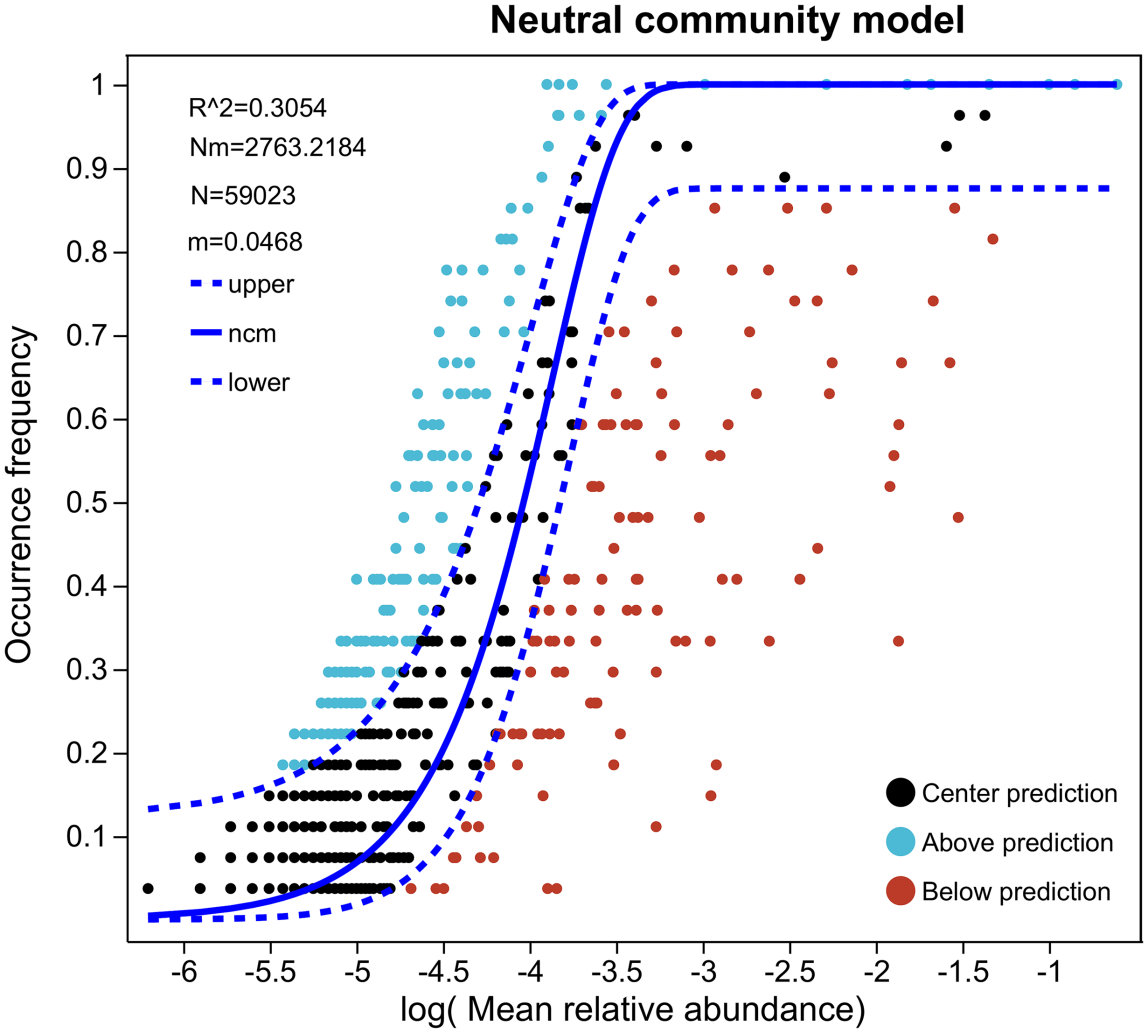
Predicting the species abundance and distribution frequency of gut microbiota based on the neutral community model (NCM). The frequency of occurrence in the C, G, Z, and ZG groups was predicted by gut microbiota. Whereas the solid blue line indicates the best fit to the NCM, the dashed blue line displays 95% confidence intervals around the NCM hypothesis. To show OTUs that happen more or less frequently than the NCM predicted, different colors are employed. *R*^2^ displays how well this model fits.

The NCM effectively represented the shift in the relative abundance and frequency of occurrence of the gut microbiota in the four groups (*R*^2^ = 0.305, m = 0.0468) (Figure 3). If G, Z, C, and ZG groups were the sole ones driving microbiota dynamics, then random patterns in microbial co-occurrence and the autocorrelation of G, Z, C, and ZG groups should be the main features of microbiota, according to neutral theories (Figure 3). There were N = 59,032 sequences for gut samples, with the gut having the lowest Nm-value (Nm = 2763.22) (Figure 3). The findings showed that the gut microbiota did not precisely follow the neutral model and that deterministic mechanisms, rather than stochastic ones, were more likely to have an impact on their formation (Figure 3).

### 3.4 Microbiota assembly processes and enterotype analysis

**Figure 4:**
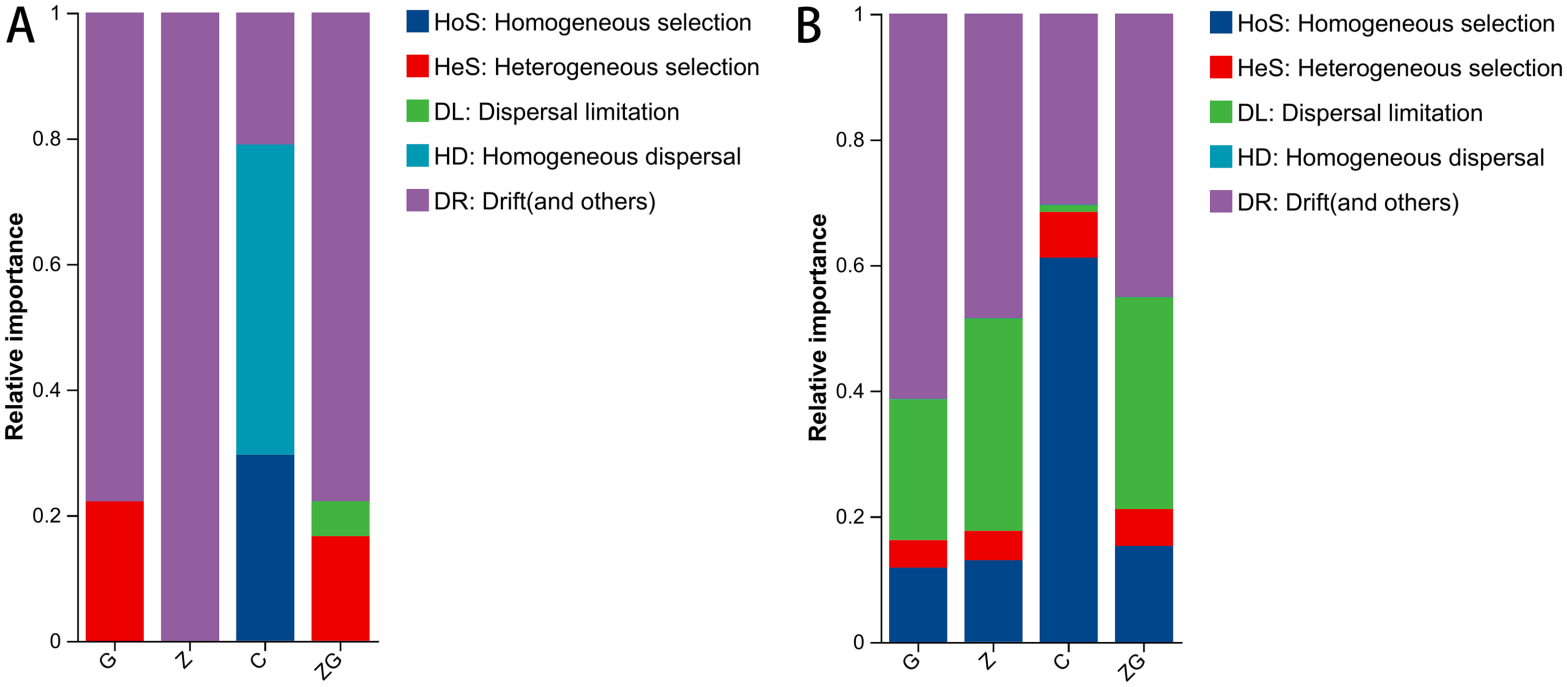
Comparative analysis of the relative importance of ecological processes across different groups using βNTI and iCAMP patterns. Based on the βNTI and the RCBray, deterministic and stochastic processes are classified into five fundamental ecological processes. A microbiota’s βNTI **(A)** and iCAMP **(B)** in connection to important factors for stochastic processes. The percentage stacked bar chart of OTU relative abundance illustrates the relative importance of these five ecological processes in the C, G, Z, and ZG groups.

We measured bacterial community assemblies using a null model analysis and discovered that there were stochastic processes associated with G, Z, C, and ZG in four ecosystems (Figure 4A). The four sample treatments’ βNTI patterns differed significantly from one another. Specifically, drift (and others) continuously dominated the microbiota, accounting for 77.78, 100, 20.99, and 77.78% of the relative significance in G, Z, C, and ZG, respectively (Figure 4A). The iCAMP patterns varied significantly across the four sample treatments (Figure 4B). The microbiota assembly that was constantly dominated by drift (and others) was responsible for 61.30% of the relative importance in the G group, 48.46% in the Z group, 30.37% percent in the C group, and 45.10% in the ZG group (Figure 4B).

The study showed that the most of the G group belong to the *Eubacterium_1* type, majority of Z group were of the *Legionella* type, C group leaned strongly to the Eubacterium type, while those of ZG group were more likely to be of the *Aurantimicrobium*, and *Camobacterium* type (Supplementary Figure S5).

Hierarchical clustering analysis at the OTUs level revealed that the gut microbiota from the 27 samples (tadpoles) could be divided into four groups (Supplementary Figure S6). OTU154 was mostly found in groups C and G, OTU208 is prominent in groups Z and ZG, particularly in Z, and OTU214 was primarily prevalent in groups C and ZG, with a notable proportion in C (Supplementary Figure S6). The tight clustering of Group C samples indicated a significant degree of commonality in their microbial makeup (Supplementary Figure S6). Tight clustering was also shown in Group G data, indicating comparable microbial compositions (Supplementary Figure S6). Samples from groups Z and ZG, on the other hand, were branched into distinct clusters, highlighting the variation in their microbial compositions (Supplementary Figure S6).

### 3.5 Composition, variations and indicator taxa of gut microbiota

**Figure 5:**
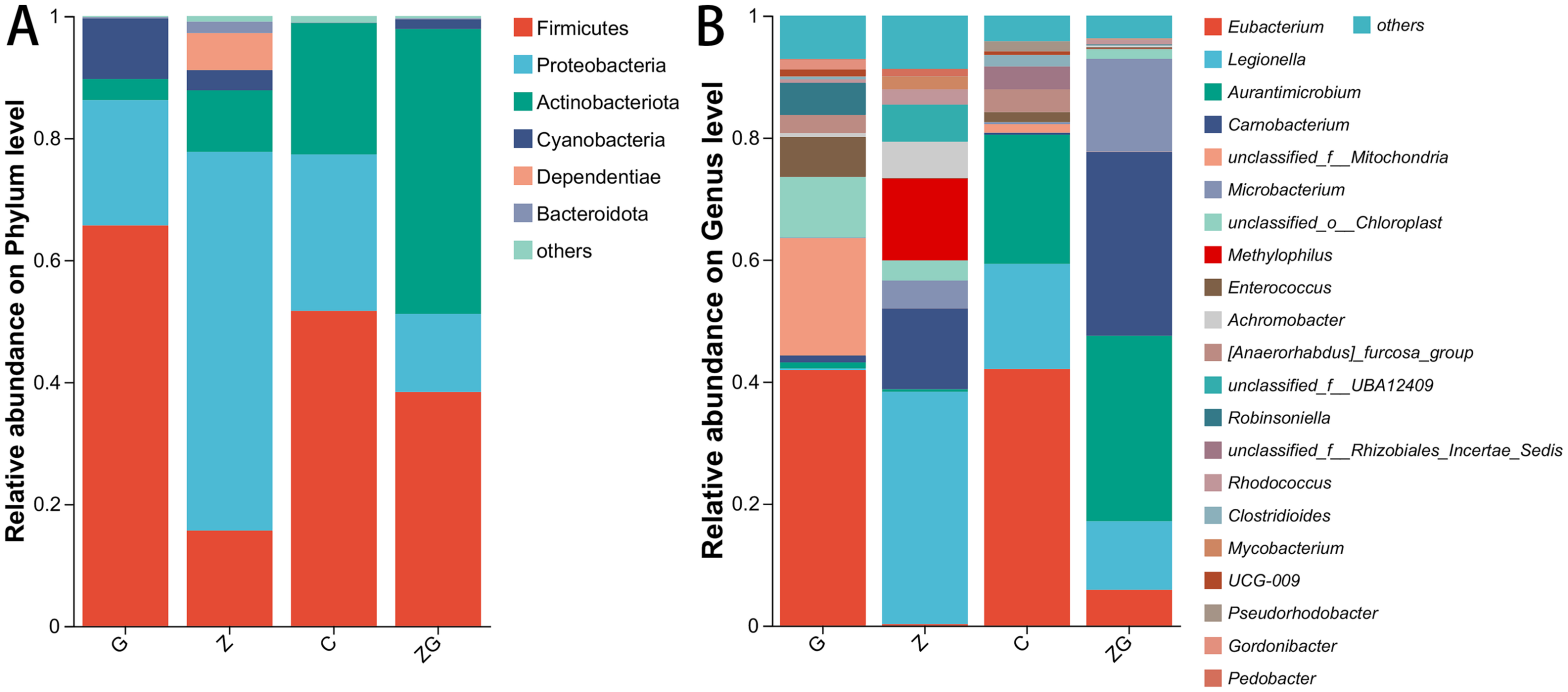
Relative abundance of gut microbial composition at phylum and genus levels in various treatment conditions. Depicting the assessment of gut microbiota at the phylum **(A)** and genus **(B)** levels. The microbiota and their relative abundances and the trends of changes in the C, G, Z, and ZG groups are displayed. Only those phylum and genus that exhibit a relative abundance greater than 1% in at least one sample. The different colored bars represent different phylum or genus, and the length of each bar indicates the proportion.

The primary phyla of gut microbiota across the C, G, Z, and ZG treatment groups included Actinobacteriota, Bacteroidota, Cyanobacteria, Dependentiae, Firmicutes, and Proteobacteria (Kruskal–Wallis H test with FDR correction, adjusted *p* < 0.01; Figure 5A). Among the 29 phyla, Actinobacteriota, Bacteroidota, Cyanobacteria, Chloroflexi, Dependentiae, Firmicutes, Fusobacteriota, Myxococcota, Planctomycetota, Proteobacteria, Patescibacteria, Verrucomicrobiota, and unclassified_d__Bacteria (Kruskal–Wallis H test with FDR correction, adjusted *p* < 0.01; Figure 5A). The dominant genera varied across groups: the C group had *Aurantimicrobium*, *Eubacterium*, *Legionella*, and *unclassified_f__Rhizobiales_Incertae_Sedis*; the G group had *Eubacterium*, *Enterococcus*, *unclassified_o__Chloroplast*, and *unclassified_f__Mitochondria*; the Z group had *Achromobacter*, *Carnobacterium*, *Legionella*, and *Methylophilus*; and the ZG group had *Aurantimicrobium*, *Carnobacterium*, *Legionella*, and *Microbacterium*. The gut microbiota, comprising 439 genera, showed significant differences, with 130 genera (such as *Acidovorax*, *Akkermansia*, *Bacillus*, *Candidatus_Paracaedibacter*, *Dietzia*, *Monoglobus*, *Methylocystis*, *Neochlamydia*, *Pediococcus*, *Succinispira*, *Thermobacillus, Thermoactinomyces*, *Turicibacter*, *Thermoclostridium*, and *unclassified_f__Parachlamydiaceae* and so on) being notably abundant (Kruskal–Wallis H test with FDR correction, adjusted *p* < 0.01; Figure 5B, Supplementary Figure S7).

**Figure 6:**
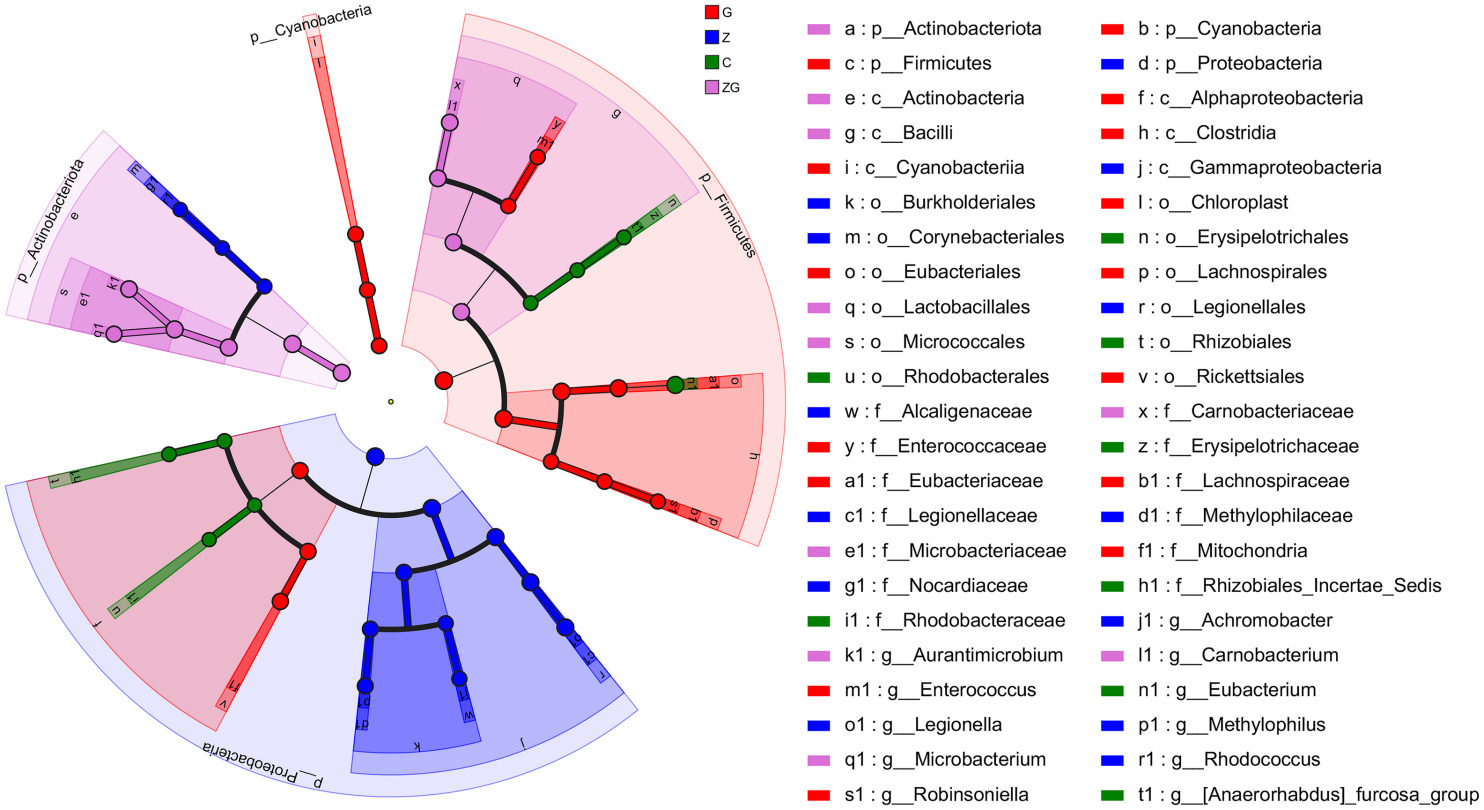
Linear discriminant analysis effect size (LEfSe)-based from phylum to genus levels differential analysis reveals gut microbiota variations across four groups. Using the LDA score (LDA > 4) to assess contributes to the observed differences from phylum to genus. The results are visualized in a phylogenetic tree, highlighting variations in the gut microbiota among different groups, certain phylum or genus may play crucial roles in environmental changes. Colored nodes represent species significantly enriched in each group, with node size indicating relative abundance.

At the phylum level, LEfSe analysis indicated an enrichment of Firmicutes and Cyanobacteria in the G group, Proteobacteria in the Z group, and Actinobacteriota in the ZG group. At the genus level, the C group had *Eubacterium* and *[Anaerorhabdus]_furcosa_group*; and *Aurantimicrobium*, *Carnobacterium*; the G group had *Robinsoniella* and *Enterococcus*; the Z group had *Achromobacter*, *Legionella*, *Methylophilus*, and *Rhodococcus*; and *Microbacterium* in the ZG group. (LDA > 4, *p* < 0.05; Figure 6).

Indicator taxa analysis identified 41 significant indicator taxa (*p* < 0.05; Supplementary Table S7). Among them, 10 were associated with group C (e.g., *Lactobacillus kefiranofaciens, Pseudorhodobacter,* and *Bosea*); 16 were associated with group G (e.g., *Christensenella minuta*, *Gordonibacter massiliensis*, and *Robinsoniella peoriensis*); and 14 were associated with group Z (e.g., *Mycobacterium tuberculosis*, *Pseudomonas syringae pv. actinidiae*, and *Streptococcus cristatus*), and one were associated with group ZG (unclassified Beijerinckiaceae).

### 3.6 BugBase and KEGG analysis

**Figure 7:**
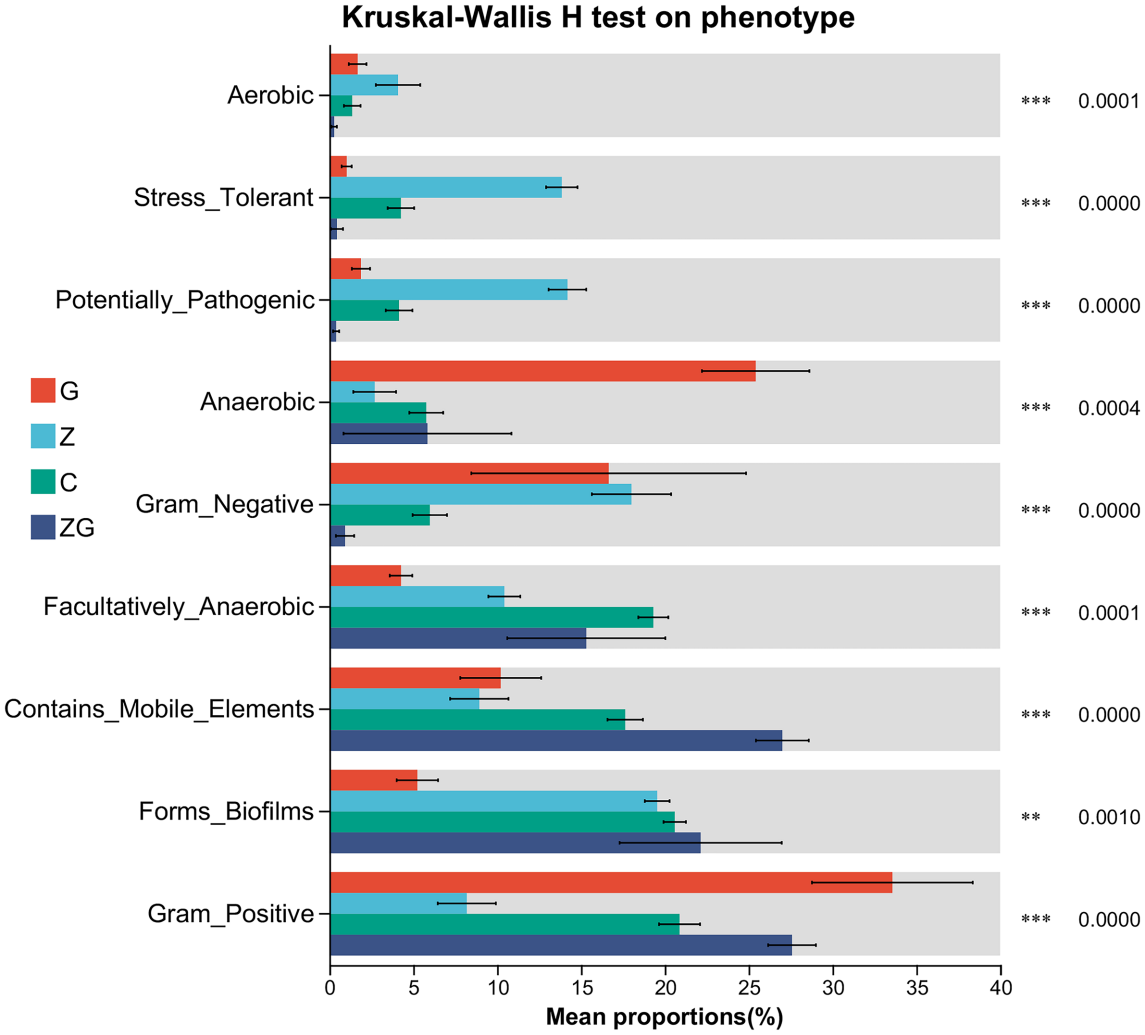
Comparison of gut microbial phenotypes in C, G, Z, and ZG group samples using BugBase phenotype prediction. BugBase phenotype prediction can identify the high-level phenotypes present in the C, G, Z, and ZG group samples, enabling gut phenotype prediction. Through contribution analysis and differential analysis, it is possible to further understand the changes in gut microbial phenotypes after treatment. Showing the proportion of different phenotype categories in each group. Significance levels are indicated: **, and *** for *p*-values falling in 0.001 < *p* < 0.01, and *p* < 0.001, respectively.

We analyzed nine phenotypes from BugBase (including Aerobic, Anaerobic, Contains_Mobile_Elements, Facultatively_Anaerobic, Forms_Biofilms, Gram_Negative, Gram_positive, Potentially_Pathogenic, Stress_Tolerant), and showed significant differences in all nine phenotypes across the different groups (Kruskal–Wallis H test, *p* < 0.05; Figure 7, Supplementary Figure S8). In the KEGG analysis, significant differences among treatment groups were observed in 43 KEGG pathways (Kruskal–Wallis H test with FDR correction, Nemenyi post-hoc test, adjusted *p* < 0.05; Supplementary Figure S9). Notable pathways included those related to immune system, metabolism (such as lipid, amino acid, and carbohydrate), and cellular processes (e.g., replication and repair, signal transduction).

## 4 Discussion

### 4.1 Alpha diversity and MDI

Exposure to ZnO NPs or glyphosate significantly increased the MDI in the Z and G groups compared with the control group, indicating the disruption of the gut microbiota balance by these chemicals (Han et al., 2025). Specifically, the Z group, exposed solely to ZnO NPs, showed the highest MDI, which may be related to the physicochemical properties of ZnO NPs, such as the generation of oxidative stress, release of heavy metal ions, or direct damage to microbial cell structures, leading to widespread negative effects on the gut microbiota (Gomes et al., 2023; Murthy et al., 2022; Xie et al., 2022). The MDI of the G group, exposed only to glyphosate, showed a slight increase, but remained relatively low, possibly due to glyphosate’s primary mechanism of inhibiting the growth of specific bacteria, which mildly affected the microbiota (Chen et al., 2022; Burgos-Aceves et al., 2024; Zhu et al., 2022). The ZG group was simultaneously exposed to ZnO NPs and glyphosate, its MDI value was higher than that of the control group and the G group exposed solely to glyphosate, but lower than the Z group. This suggests that the combination between ZnO nanoparticles and glyphosate did not yield the anticipated synergistic effect, likely due to the differing mechanisms of action of the two substances, leading to their independent impacts on the intestinal microbiota through distinct pathways, and failing to complement or enhance the overall effect (Burgos-Aceves et al., 2024; Montero et al., 2022). These results highlight the importance of further research on the impact of environmental pollutants on amphibian gut microbiota, especially given the widespread use of NPs and pesticides in the current environment, which may have long-term effects on ecosystem health and biodiversity (Annamalai et al., 2024; Chen et al., 2022; do Amaral, 2019).

In this study, the phenomenon of high MDI and high Shannon index may be caused by multiple factors. MDI reflects the health status and balance of the gut microbiota, primarily focusing on the ratio of beneficial to harmful bacteria, while the Shannon index measures the community diversity, including species richness and evenness (Liu et al., 2020; Sun et al., 2021). This means that even if the microbiota is imbalanced overall, the replacement of species and the addition of new species may still lead to high diversity (Huang et al., 2021). Furthermore, pollutants such as ZnO NPs may have selective effects on different microorganisms, inhibiting some beneficial bacteria while promoting opportunistic pathogens or microorganisms with stronger adaptability, thereby altering the original microecological balance (Wu et al., 2023; Kakakhel et al., 2023). The physical and chemical properties of ZnO NPs (such as small size and high reactivity) may also induce changes in the microenvironment (such as alterations in pH and redox status), which can cause certain microorganisms to increase in number due to their better adaptation to the new environment (Anders et al., 2018; Sun et al., 2024; Jayaseelan et al., 2014). Although this increases diversity, it does not necessarily reflect a healthy microbiota (Wu et al., 2023). Therefore, the coexistence of high MDI values and high Shannon indices may indicate that while more microbial species appear, the microbiota structure may trend toward an increase in harmful bacteria (Han et al., 2025). This complex interaction suggests that when assessing the ecological risks and health impacts of environmental pollutants, it is necessary to integrate various ecological and microbiological indicators to fully understand their comprehensive effects on microbiota (Chen et al., 2022; Boccioni et al., 2021; Villatoro-Castañeda et al., 2023).

### 4.2 Beta diversity

In this study, Bray–Curtis and weighted UniFrac analyses showed significant differences in tadpole gut microbiota among groups, revealing combined effects of ZnO NPs and glyphosate. Specifically, the distance between the C group and the G group was the smallest, indicating that glyphosate had a relatively mild effect on the gut microbiota (Chen et al., 2022). The G and ZG groups were similar, indicating that the combined exposure to glyphosate and ZnO NPs may not have markedly amplified the effects of glyphosate, potentially due to an interaction or their counteracting effects (Burgos-Aceves et al., 2024; Montero et al., 2022). However, the Z group was significantly farther from the other groups, indicating that ZnO NPs had the greatest impact on the gut microbiota (Wu et al., 2023; Murthy et al., 2022; Sun et al., 2024; Motta and Guerra, 2023). This finding suggests that ZnO NPs may significantly affect the gut microbiota through strong ecological toxicity, potentially threatening amphibian health and adaptability (Wu et al., 2023; Chen et al., 2022; Gomes et al., 2023; Long et al., 2023; Bordin et al., 2024). ZnO NPs may affect the intestinal microbiota through two mechanisms: the accumulation of ZnO NPs in the body may lead to local oxidative stress, thereby compromising the stability of intestinal microorganisms; the physical and chemical properties of ZnO NPs may change the pH and ionic strength of the intestinal environment, thereby affecting the growth and reproduction of microorganisms. In contrast, glyphosate mostly demonstrates selective suppression of particular bacteria, resulting in a limited overall impact range (Wu et al., 2023; Chen et al., 2022). These results provide useful insights into how pollutants affect amphibian gut microbiota, specifically that ecological toxicity of ZnO NPs may drastically alter gut microbiota structure, posing potential risks to animal health (Motta and Guerra, 2023; Bordin et al., 2024; Adamovsky et al., 2018). These findings provide key data for evaluating the long-term effects of NPs and pollutants on ecosystems, highlighting their potential risks to amphibian health and adaptation (Wu et al., 2023; Chen et al., 2022; Chen et al., 2022; Edge et al., 2013; Boccioni et al., 2021).

### 4.3 Composition

LEfSe analysis revealed distinct microbial enrichments at phylum and genus levels across different treatment groups. The enrichment of *Robinsoniella* and *Enterococcus* in group G indicates that glyphosate exposure may alter the intestinal microenvironment and the structure of available nutrient substrates, resulting in a relative dominance of microbiota associated with protein and organic matter metabolism (Yang et al., 2024; Emerson and Woodley, 2017; Ashkar and Wu, 2023). Simultaneously, in the context of disturbance, genera with opportunistic traits may proliferate, so potentially destabilizing the community and impairing host immunological homeostasis. The enrichment of *Achromobacter*, *Rhodococcus*, *Methylophilus* and *Legionella* in group Z suggests that ZnO NPs exert selective pressure, favoring stress-tolerant and environmentally adaptable species, thus driving the community toward “tolerance” recombination. This may alter the original symbiotic network structure, potentially disrupting intestinal homeostasis (Han et al., 2025; Lv et al., 2023). The enrichment of *Methylophilus* may indicate a transition in metabolic pathways associated with one-carbon metabolism, whereas the emergence of *Legionella* highlights that environmental source bacteria achieve higher abundance in the intestine, suggesting alterations in the host’s “filtration and selection” of exogenous microorganisms, which may affect barrier or local immune regulation (Wu et al., 2023; Wang et al., 2023; Yang et al., 2024). The proliferation of *Aurantimicrobium*, *Carnobacterium* and *Microbacterium* in the combined treatment group (ZG group) indicates that the exposure induces community reorganization and niche redistribution distinct from single exposure. The enrichment of environmentally-associated taxa may reflect shifts in colonization opportunities or host selection processes, while *Carnobacterium*, as a lactic acid bacterium, may play a role in maintaining local homeostasis (Chen et al., 2022; Boccioni et al., 2021; Villatoro-Castañeda et al., 2023). In summary, distinct exposure types are associated with different microbial community reassembly pathways, and may alter the microbial ecological functions related to host metabolism and immunity (Chen et al., 2022; Motta et al., 2018; Motta et al., 2024). Furthermore, the potential bidirectional exchange between the tadpole gut and aquatic microbiota, along with the increased relative abundance of environmentally associated taxa under combined exposure, suggests alterations in exogenous microbial input or host filtering processes. These changes may disturb host-environment microbial connectivity and recolonization dynamics, providing insight into how pollutants affect host-environment microbial cycling (do Amaral DF, 2019; Mann et al., 2009). However, the lack of sequencing data from rearing water and feed as environmental background controls precludes confident source attribution for environment-associated taxa. Future work will incorporate water and feed samples as negative controls to strengthen source attribution.

IndVal analysis identified 41 significant indicator genera (*p* < 0.05), eight of which were assigned to species level. Based on prior literature, some indicator bacteria are associated with gut homeostasis or potential benefits: *Lactobacillus kefiranofaciens* has been reported to have immunomodulatory and probiotic potential relevant to gut health (Georgalaki et al., 2021); *Christensenella minuta* has been associated with metabolic health and proposed as a next-generation probiotic candidate (Ignatyeva et al., 2024); *Gordonibacter massiliensis* has been associated with the metabolism of dietary polyphenols (e.g., ellagic acid) and the production of urolithins with anti-inflammatory and antioxidant bioactivities (García-Villalba et al., 2020); *Streptococcus cristatus* has been reported to suppress virulence gene expression in pathogenic bacteria in microbial interaction studies, showing potential protective effects (Ho et al., 2017). It should be emphasized that these associations likely reflect selective shifts in community composition or compensatory responses under exposure stress, rather than indicating an overall beneficial effect of exposure. These results suggest that exposure may not only reshape community structure but also alter the composition of host health-related functional taxa. The risk-associated indicator bacteria were primarily detected in groups Z and G. *Robinsoniella peoriensis* has been reported as an opportunistic pathogen and was identified as an indicator species for group G in this study, suggesting that glyphosate exposure may be associated with the enrichment of opportunistic pathogen-associated taxa (Ioannou et al., 2024). *Mycobacterium tuberculosis* and *Pseudomonas syringae pv. actinidiae* were recognized as important pathogens in human and plants, respectively (Warner et al., 2025; Wan et al., 2025). However, in 16S amplicon analyses, species-level assignments are limited by taxonomic resolution and high sequence similarity among closely related taxa (Janda and Abbott, 2007). Therefore, these “species-level” indicator signals are more likely to reflect abundance shifts in environmentally derived or opportunistic taxa closely related to the target species, and should not be directly interpreted as evidence that tadpoles were infected with the corresponding pathogen. Overall, the indicator taxa results suggest potentially risk-associated community shifts in the exposure groups (especially Z and G), which require validation using higher-resolution approaches (e.g., metagenomics, qPCR-based quantification, or culture-based isolation).

### 4.4 Function

Since BugBase was a predictive tool used in 16S analysis, we interpret the findings of our study with caution. According to BugBase analysis, there were significant phenotypic differences in the gut microbiota among all groups, with nine phenotypes showing variation. Significant differences were observed in both anaerobic and aerobic pathways, indicating that exposure to environmental treatments influenced the microbial metabolism based on available oxygen (Lu and Imlay, 2021). Notably, the Contains Mobile Elements and Stress-Tolerant phenotypes showed substantial variations, suggesting that microbial resilience and adaptability were affected by environmental stressors, such as glyphosate and ZnO NPs (Li et al., 2023). The combination treatment group exhibited shifts in microbial adaptability and a potential increase in horizontal gene transfer (Husnik and McCutcheon, 2018). The Facultatively Anaerobic phenotype was also notably impacted, reflecting the microbiota’s flexibility in adapting to fluctuating oxygen levels (Solak et al., 2024). Furthermore, significant differences in Biofilm Formation and Potentially Pathogenic bacteria were observed, particularly in the glyphosate and combination groups, suggesting that these treatments may enhance microbial resistance to environmental stresses and increase the potential for pathogenicity (Motta et al., 2024). Lastly, changes in the balance of Gram_Positive and Gram_Negative bacteria were observed, with the combination treatment group showing an increased abundance of Gram_negative microbes (Fanin et al., 2019). These findings highlight how environmental treatments can significantly alter microbial phenotypes, potentially affecting microbial resilience, pathogenic potential, and overall gut microbiota stability, which could influence host health, immune function, and disease susceptibility (Xavier et al., 2024).

### 4.5 Significance

This study investigates the impact of ZnO NPs and glyphosate on the gut microbiota structure of *Rana dybowskii* tadpoles, with significant ecological toxicological implications. In the context of increasing environmental pollution, the effects of pollutants such as nanoparticles and pesticides on ecosystems have become a global concern (Wu et al., 2023; Chen et al., 2022). While previous studies have explored the impact of external pollutants on microbial communities, the effects of ZnO NPs and glyphosate on amphibian gut microbiota remain insufficiently studied (Boccioni et al., 2021; Bordin et al., 2024). This research fills this gap. The results show that both individual and combined exposures significantly altered the diversity of the gut microbiota, especially in terms of changes in the abundance of dominant bacterial species (Wu et al., 2023; Chen et al., 2022). The impact of pollutants on microbiota was primarily driven by deterministic processes, indicating that pollutants modify microbial growth conditions and metabolic pathways, thereby directionally altering microbiota structure (Sun et al., 2022). This finding provides a new perspective on how pollutants may indirectly affect biological health through changes in microbiota (Wu et al., 2023; Boccioni et al., 2021; Villatoro-Castañeda et al., 2023). Additionally, the study emphasizes the potential risks associated with gut microbiota dysbiosis, disrupted metabolic, immune, and disease-related pathways, which could increase the susceptibility of tadpoles to diseases (Yang et al., 2024; Emerson and Woodley, 2017; Ashkar and Wu, 2023). The functional disruption highlighted in this study is of significant value in assessing the impact of environmental pollution on biological health (Wu et al., 2023). More importantly, the dysbiosis caused by pollutants could weaken amphibians’ physiological health, potentially impacting species survival and ecosystem stability (Chen et al., 2022; Boccioni et al., 2021; Villatoro-Castañeda et al., 2023). As environmental pollution issues intensify, it is increasingly urgent to assess the long-term effects of pollutants on ecosystem health (Knutie et al., 2018; Wu et al., 2023). This study offers a new perspective for ecological toxicology research and provides scientific evidence for the formulation of environmental protection policies (Wu et al., 2023).

## 5 Conclusion

This study demonstrates that exposure to ZnO NPs, glyphosate, and their combination significantly disrupts the gut microbiota of *R. dybowskii* tadpoles. Elevated MDI and altered alpha diversity in the ZnO and combined ZG groups indicate a pronounced imbalance in microbial communities, potentially compromising tadpole health and resilience. Beta diversity analyses further confirmed distinct microbial assemblages across treatment groups, highlighting the specific impacts of each pollutant and their synergistic effects. Shifts in dominant microbial taxa and functional pathways suggest alterations in metabolic processes and immune functions, which may increase susceptibility to diseases and affect overall physiological stability. These findings underscore the critical need for comprehensive risk assessments of environmental pollutants on amphibian gut microbiota. Future research should explore long-term ecological consequences, underlying molecular mechanisms, and potential mitigation strategies to protect amphibian populations from escalating environmental threats.

## Data Availability

The datasets presented in this study can be found in online repositories. The names of the repository/repositories and accession number(s) can be found in the article/Supplementary material.

## Footnotes

1
https://mothur.org/wiki/calculators/

2
https://qiime2.org
