# Supplementary material for: Assessing the individual and combined effects of ZnO nanoparticles and glyphosate on the gut microbiota of tadpoles
Source: Front Microbiol. 2026 Mar 26;17:1751143. doi: 10.3389/fmicb.2026.1751143 (PMC13062192; doi:10.3389/fmicb.2026.1751143)
Supplement: Supplementary file 1 [file Supplementary_file_1.docx]

**Supply materials**

**Assessing the Individual and Combined Effects of ZnO Nanoparticles and Glyphosate on the Gut Microbiota of *Rana dybowskii* Tadpoles**

Xue-dong Han^a^, Peng Guo^a^, Qing Tong^a^, Hong-sheng Yang^a^, De-cai Liu^a^*

a College of Biology and Agriculture, Jiamusi University, Jiamusi, 154007, China

*Correspondence:

De-cai Liu, [dcailiu@126.com](mailto:dcailiu@126.com)

Word count: 6300

Number of figures: 7



**Figure S1** Intensity-weighted hydrodynamic size distributions of ZnO nanoparticles measured by dynamic light scattering (DLS).

DLS intensity-weighted size distribution profiles of ZnO NPs . Panels A–C represent three independent measurements. The inset in each panel reports the Peak 1 diameter (d.nm) and PDI; Peak 1 accounted for 100% intensity in all runs, indicating a unimodal intensity distribution


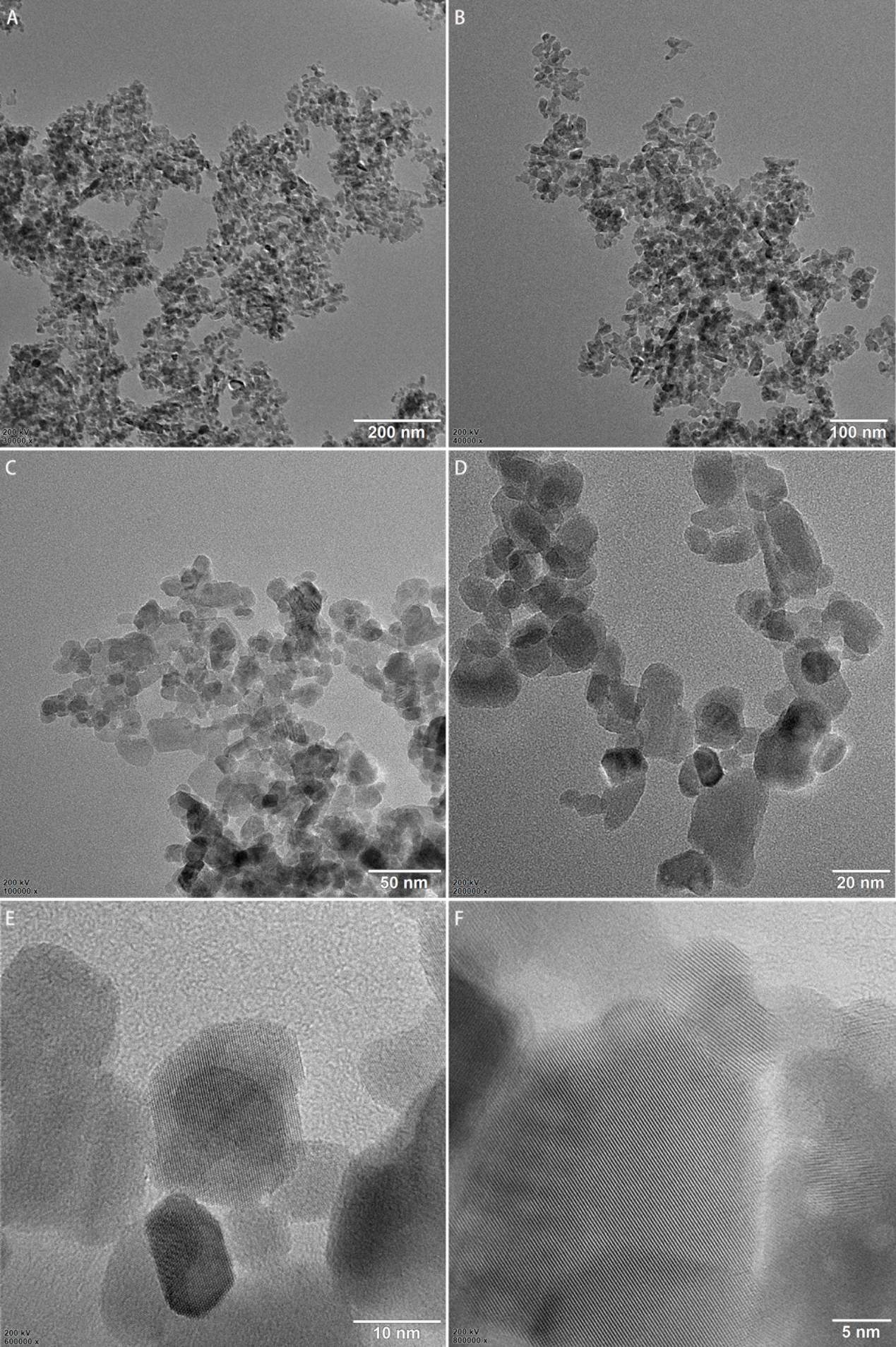


**Figure S2** Transmission electron microscopy (TEM) and high-resolution transmission electron microscopy (HRTEM) characterization of ZnO nanoparticles.

Transmission electron microscopy (TEM) images of ZnO nanoparticles at increasing magnification (A–D) and lattice-resolved high-resolution TEM (HRTEM) images (E–F) acquired on the same instrument.


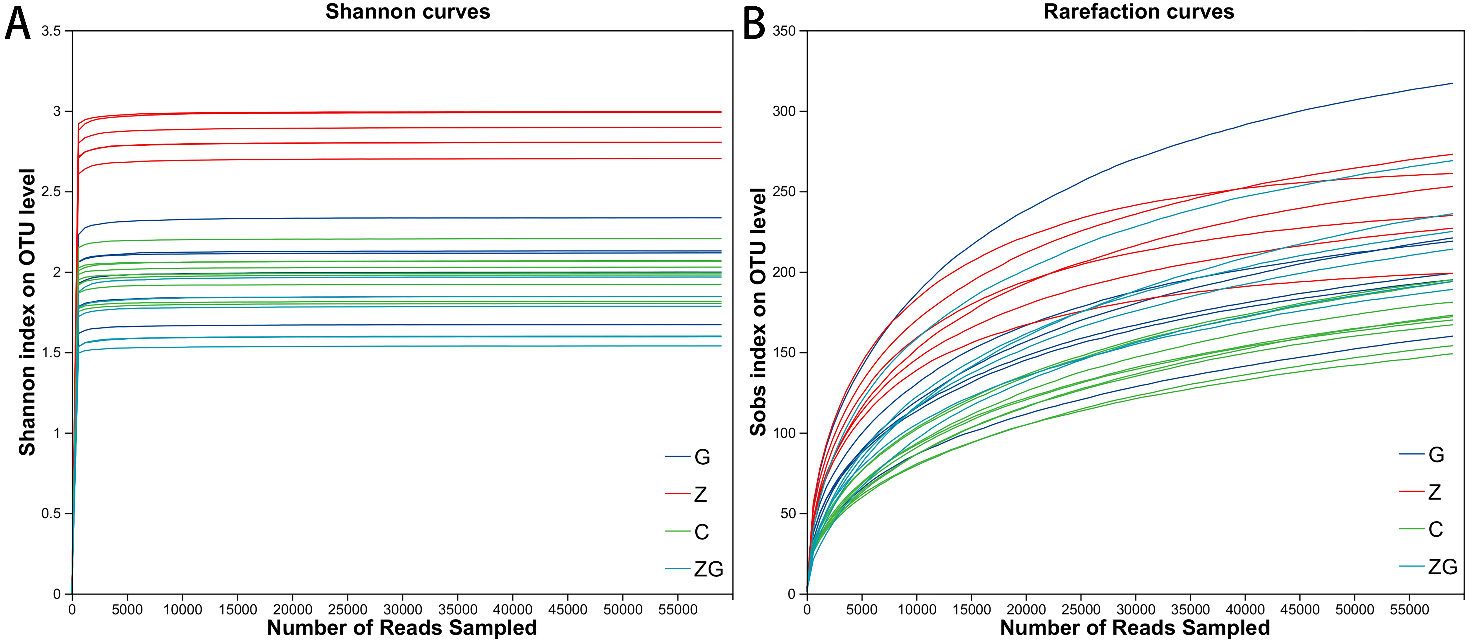


**Figure S3** Shannon curves and Rarefaction curves.

The Shannon curves (A) and Rarefaction curves (B) were plots of the number of OTUs as a function of the number of sequences. The Shannon curves reflect the microbial diversity of the samples at different sequencing quantities.


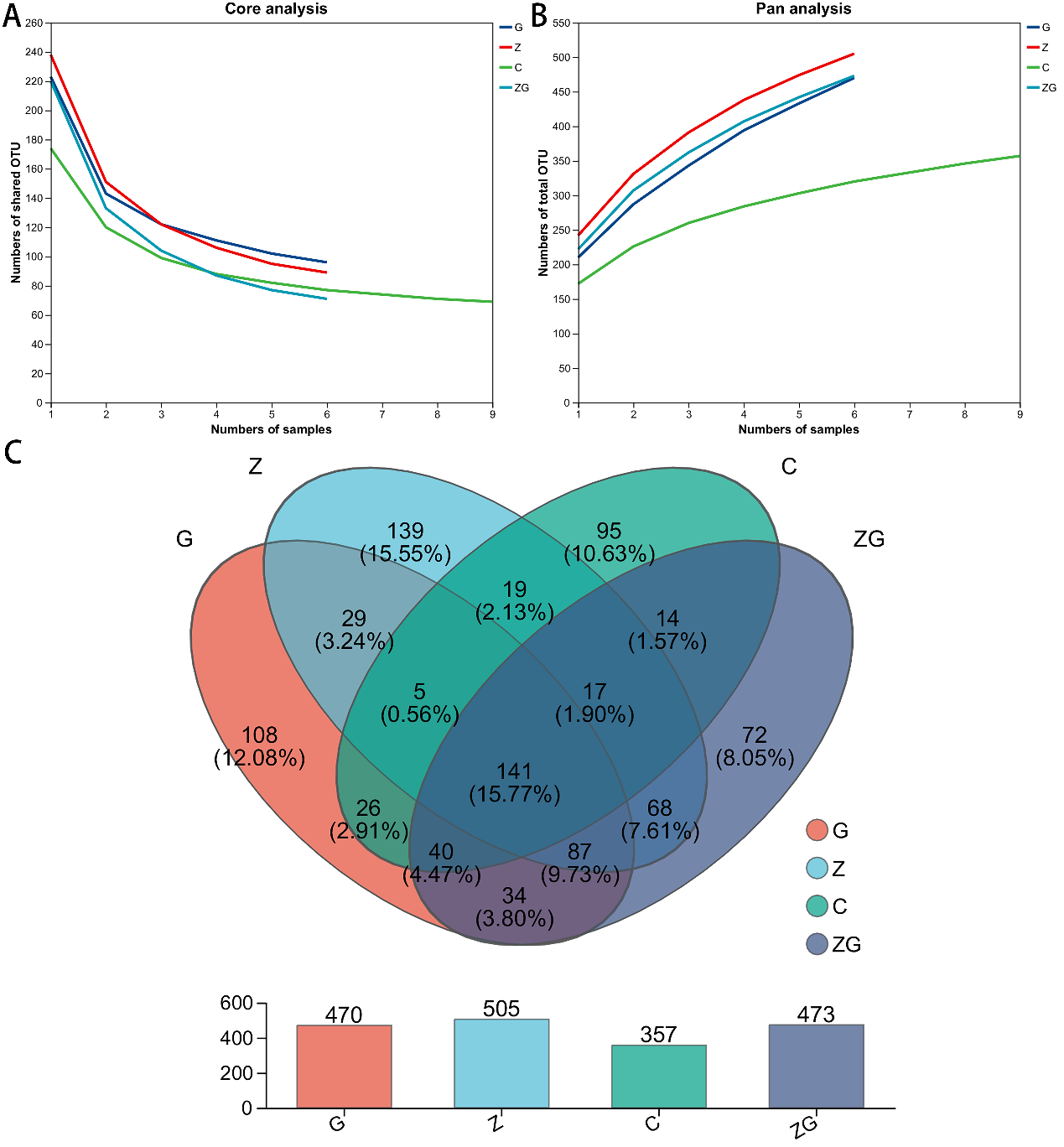


**Figure S4** Core–pan OTU diversity and overlap among four groups (G, Z, C, and ZG).

(A) Core analysis: number of shared OTUs as the number of samples increases in each group (G, Z, C, ZG). (B) Pan analysis: cumulative total OTUs as the number of samples increases in each group. (C) Venn diagram showing unique and shared OTUs among the four groups (counts with percentages), with a bar chart summarizing total OTUs per group.


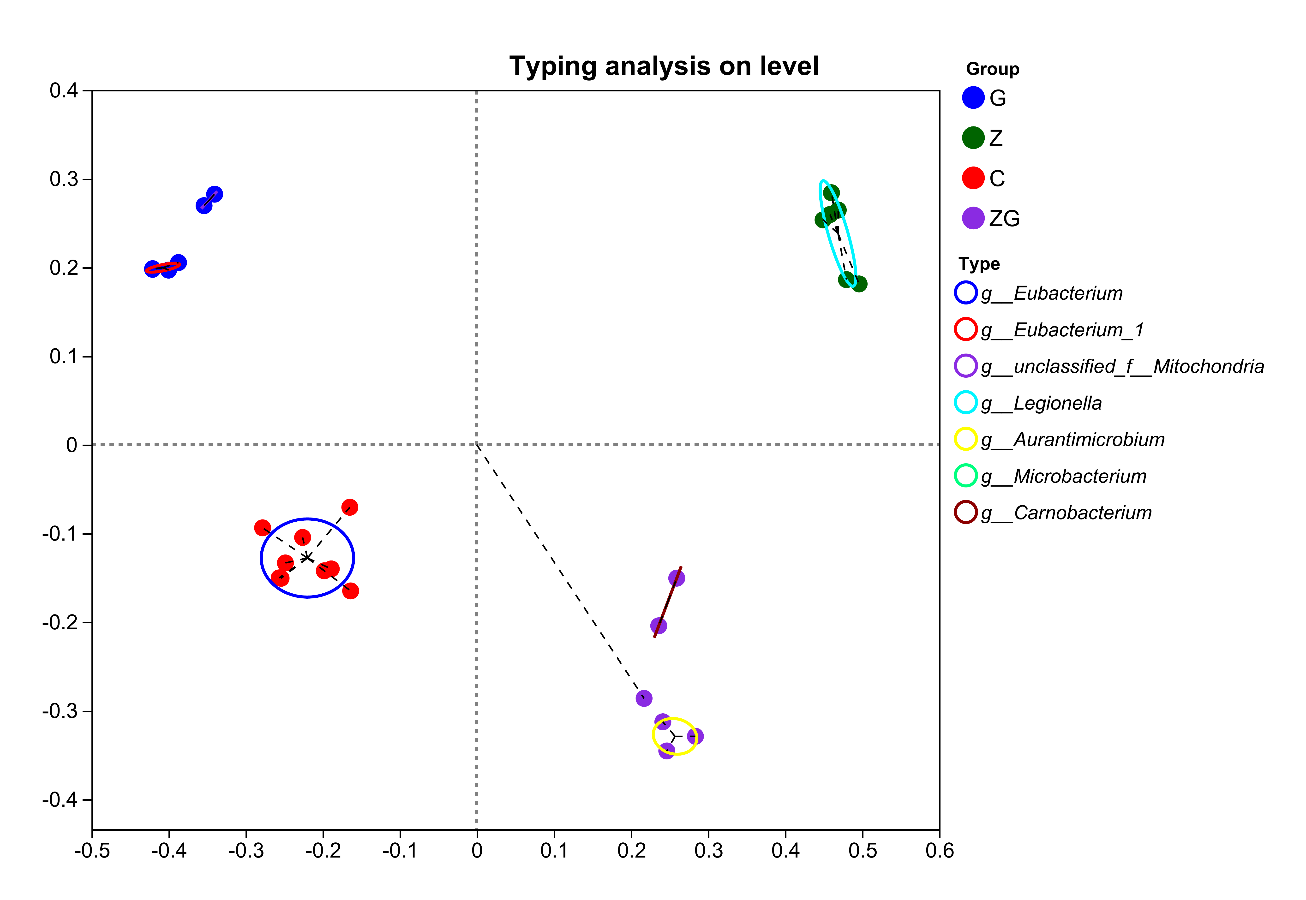


**Figure S5** The C, G, Z, and ZG groups' enterotype analysis is above the genus level.

The "Group" in the top right corner represents different sample groups; "Enterotype" indicates different classifications (only showing classification names that contain two or more samples). The area within the circle corresponds to the range that meets the credible interval.


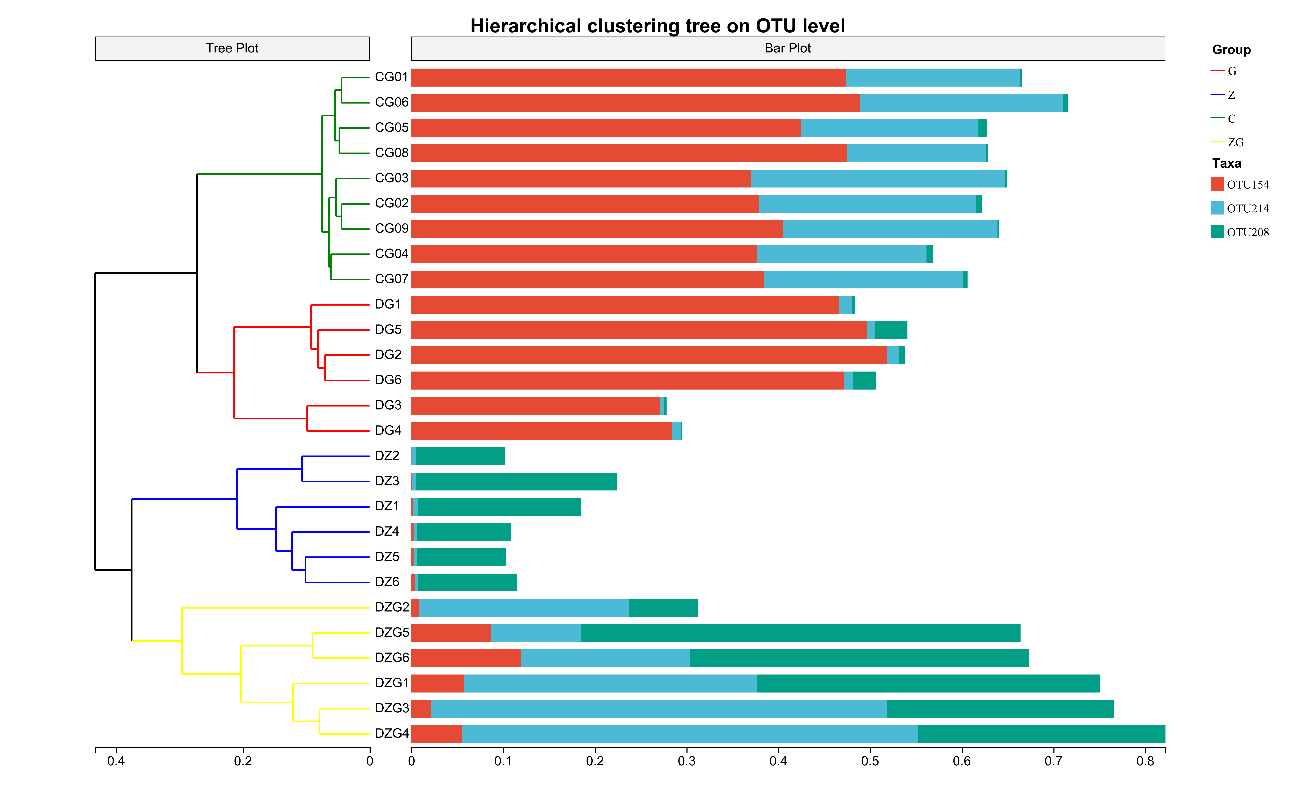


**Figure S6** Hierarchical clustering analysis reveals group structures at the OTU level.

Showing a hierarchical clustering tree alongside corresponding bar plots based on OTU level. Different color lines represent different sample groups, with each color denoting one group. The colors in the bar plots indicate the relative abundance of various OTUs within each sample. The x-axis represents the relative abundance of OTUs, and the y-axis lists the samples. The color legend next to the clustering tree indicates the groupings of the samples.


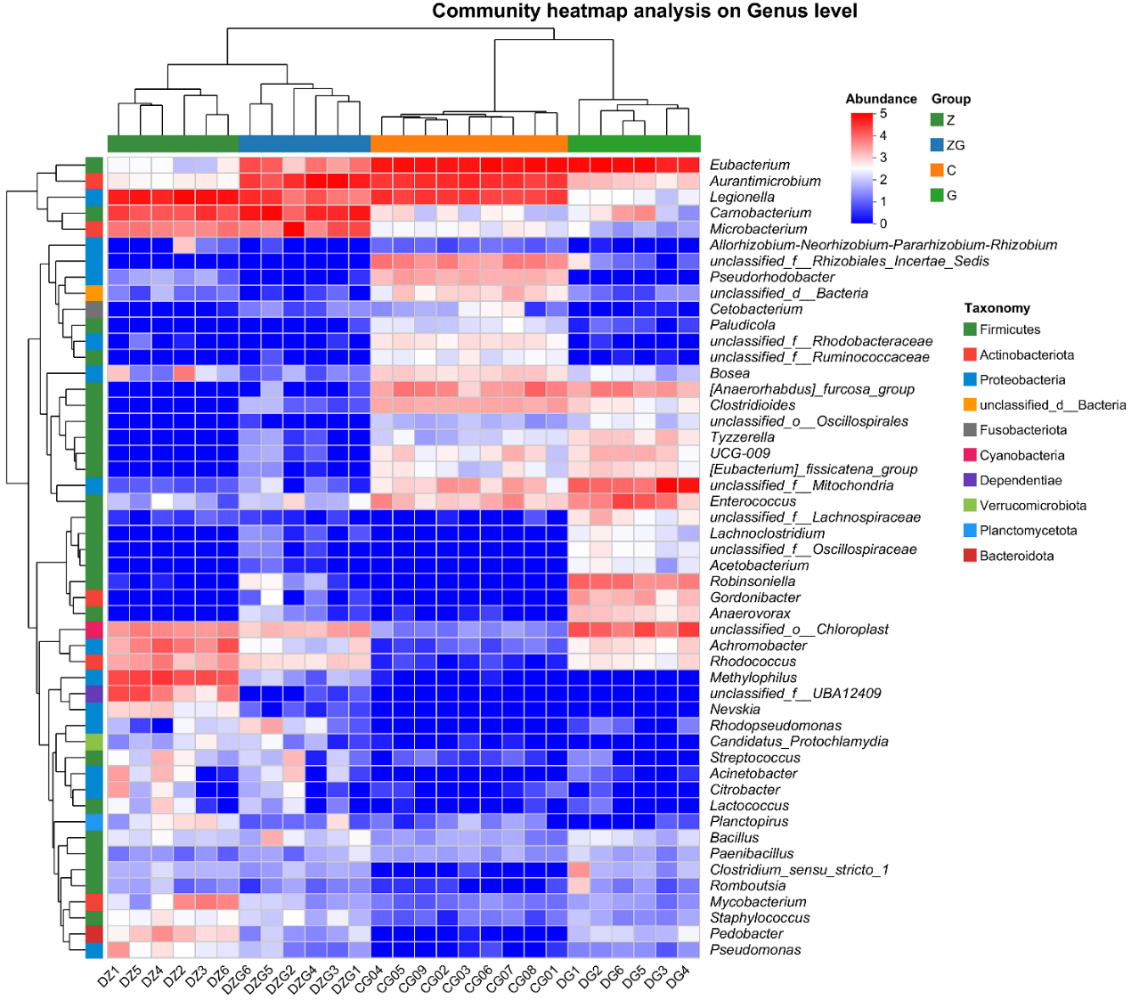


**Figure S7** Heatmap analysis of bacterial genera relative abundance in the gut microbiome across various treatment groups.

This heatmap displays the log-transformed relative abundances of bacterial genera within the gut microbiota across the C, G, Z, and ZG treatment groups. Abundance levels are indicated by a color gradient, with red representing higher abundance and blue indicating lower abundance. Hierarchical clustering has been utilized to organize samples and genera based on their abundance profiles. Additionally, a bubble plot provides further visualization of taxonomic classifications and relative abundances of genera within each group, where bubble size is proportional to abundance and color denotes the corresponding bacterial phylum.


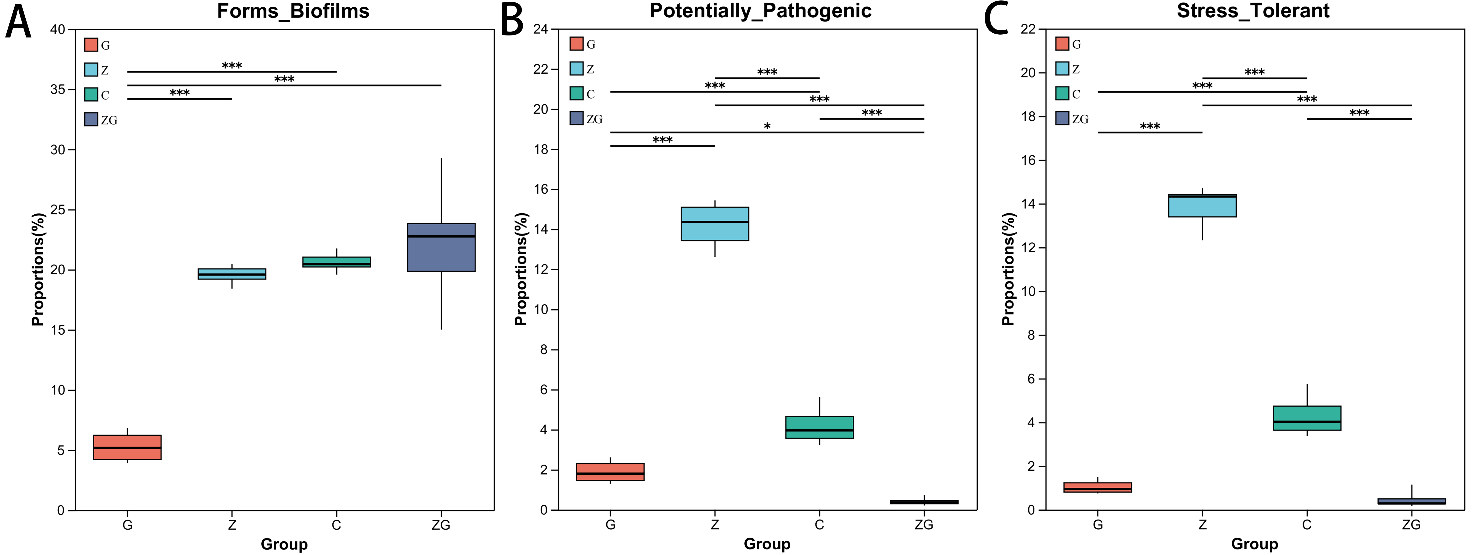


**Figure S8** Comparative analysis of bacterial phenotypic distribution among treatment groups.

This figure presents the proportions of bacterial phenotypes capable of biofilm formation (A), potential pathogenicity (B), and stress tolerance (C) across the treatment groups C, G, Z, and ZG. The y-axis quantifies the proportion of each phenotype expressed as a percentage, while the x-axis categorizes the different treatment groups. The box plots reveal the distribution and variability within each group. Statistical significances between groups are indicated by asterisks: * for *P* < 0.05, ** for *P* < 0.01, *** for *P* < 0.001.


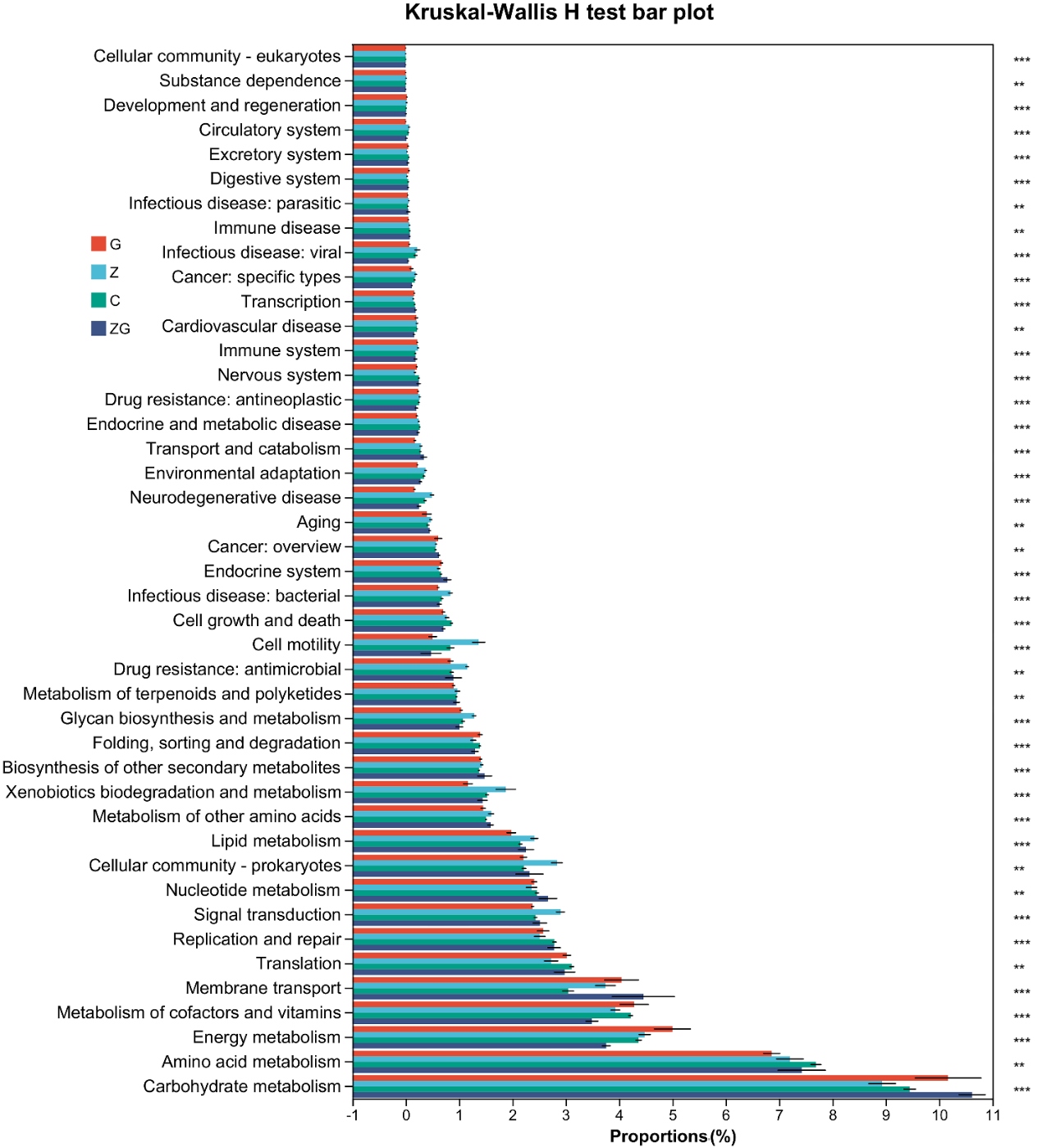


**Figure S9** Comparative analysis of predicted functional pathways in *R. dybowskii* gut microbiota.

This bar plot utilizes the Kruskal-Wallis H test to assess variations in predicted functional pathways within the gut microbiota of *R. dybowskii* across different treatment groups. Pathways are organized by secondary kyoto encyclopedia of genes and genomes (KEGG) pathways, with significant differences noted in gene abundances related to these categories. Groups are distinguished based on exposure to C, G, Z, and ZG groups. PICRUSt2 functional prediction is used to predict the functional information of gut microbiota in environmental samples, providing insights into potential microbial functional features during environmental changes through functional composition and abundance. It can predict metabolic pathways (such as KEGG) and clusters of orthologous groups (COG) associated with disease processes.

**Table S1** Sample-to-accession mapping for NCBI SRA deposition

| BioProject accession | Experimental groups | BioSample accession |
| --- | --- | --- |
| PRJNA1050064 | control group (C) | SAMN38725618 : TCG1 (TaxID: 410658) |
|  |  | SAMN38725619 : TCG2 (TaxID: 410658) |
|  |  | SAMN38725620 : TCG3 (TaxID: 410658) |
|  |  | SAMN38725621 : TCG4 (TaxID: 410658) |
|  |  | SAMN38725622 : TCG5 (TaxID: 410658) |
|  |  | SAMN38725623 : TCG6 (TaxID: 410658) |
|  |  | SAMN38725624 : TCG7 (TaxID: 410658) |
|  |  | SAMN38725625 : TCG8 (TaxID: 410658) |
|  |  | SAMN38725626 : TCG9 (TaxID: 410658) |
| PRJNA1208426 | glyphosate group (G) | SAMN46178923 : DG1 (TaxID: 749906) |
|  |  | SAMN46178924 : DG2 (TaxID: 749906) |
|  |  | SAMN46178925 : DG3 (TaxID: 749906) |
|  |  | SAMN46178926 : DG4 (TaxID: 749906) |
|  |  | SAMN46178927 : DG5 (TaxID: 749906) |
|  |  | SAMN46178928 : DG6 (TaxID: 749906) |
| PRJNA1208430 | ZnO NPs groups | SAMN46178952 : DZ01 (TaxID: 749906) |
|  |  | SAMN46178953 : DZ02 (TaxID: 749906) |
|  |  | SAMN46178954 : DZ03 (TaxID: 749906) |
|  |  | SAMN46178955 : DZ04 (TaxID: 749906) |
|  |  | SAMN46178956 : DZ05 (TaxID: 749906) |
|  |  | SAMN46178957 : DZ06 (TaxID: 749906) |
|  | glyphosate + ZnO NPs groups | SAMN46178958 : DZG1 (TaxID: 749906) |
|  |  | SAMN46178959 : DZG2 (TaxID: 749906) |
|  |  | SAMN46178960 : DZG3 (TaxID: 749906) |
|  |  | SAMN46178961 : DZG4 (TaxID: 749906) |
|  |  | SAMN46178962 : DZG5 (TaxID: 749906) |
|  |  | SAMN46178963 : DZG6 (TaxID: 749906) |

**Table S2** Summary of analysis methods, objectives, and software/packages.

| Analysis method | Purpose / output | Software / package (version) |
| --- | --- | --- |
| Shannnon and Rarefaction curves | Assess sequencing depth sufficiency and within-sample diversity among groups | mothur (version v.1.30.2); R (version 3.3.1) tool to create curves. |
| alpha diversity (Shanno, Chao, and Sobs) | Shannon:Estimate the index of microbial diversity in the sample  Chao:Indices for estimating the number of OTUs in the sample  Sobs:The actual number of observed OTUs | mothur (version v.1.30.2 [https://mothur.org/wiki/calculators/](https://mothur.org/wiki/calculators/)) |
| Alpha-diversity significance testing（FDR correction、Kruskal-Wallis H test、Tukey-Kramer） | Test differences in Shanno, Chao, and Sobs among treatment groups | The boot (version 1.3.18) and stats (version 3.3.1) packages of the R (version 3.3.1) |
| Microbiota dysbiosis index (MDI) | Quantify microbiota imbalance based on species-level profiles.. The larger the value, the greater the degree of bacterial imbalance | MetaPhlAn2 analysis; visualization using the R package vegan 2.4.3 |
| Non-metric multidimensional scaling，NMDS | Visualize the differences and clustering patterns among samples using sorting methods | Qiime (2020.2.0) was used to calculate the beta diversity distance matrix, and the NMDS analysis and plotting were performed using the vegan package (version 3.3.1) in R (version 2.4.3). |
| Neutral community model (Sloan 2006) | Evaluate stochasticity by relating relative abundance to occurrence frequency | The R language Hmisc (version 5.0.1), minpack.lm (version 1.2.3), and getopt (version 1.20.3) software packages |
| Null model analysis: βNTI | Partition deterministic vs. stochastic contributions to community assembly | The icamp package (version 1.5.12) of R language (version 3.3.1) |
| Null model analysis:iCAMP | Infer community assembly mechanisms within phylogenetic bins | The icamp package (version 1.5.12) of R language (version 3.3.1) |
| Enterotype/cluster analysis using Jensen-Shannon divergence (JSD) | Cluster samples into dominant community structure types (enterotypes) | R v3.3.1; packages ade4, cluster, clustersim |
| Venn diagram | Identify unique and shared OTUs among groups. | R v3.3.1 |
| Group differences in beta diversity (ANOSIM; Adonis/ PERMANOVA with 999 permutations) | Test treatment effects on community clustering/dispersion using UniFrac and Bray-Curtis | R vegan 2.4.3 |
| Differential abundance testing (Kruskal-Wallis H; Benjamini-Hochberg FDR) | Identify taxa with significantly different relative abundance among groups | R v3.3.1 |
| Ternary plot | Visualize distribution/interactions of dominant taxa across C, G, Z, ZG groups | R packages ggtern, ggplot2 |
| Biomarker discovery (LEfSe; LDA>4) | Identify taxa that best explain differences among groups | http://galaxy.biobakery.org/ |
| BugBase phenotype prediction | Predict high-level microbial phenotypes (e.g., aerobic/anaerobic, pathogenic potential) | https://bugbase.cs.umn.edu/index.html |
| Functional profiling (KEGG) | Predict KEGG ortholog/pathway profiles from 16S data | PICRUSt2http://huttenhower.sph.harvard.edu/galaxy |

**Table S3** Indicator species analysis (IndVal) identifying taxa associated with each group (C, G, Z and ZG).

| **Name** | **C_indicator** | **G_indicator** | **Z_indicator** | **ZG_indicator** | ***P_*value** |
| --- | --- | --- | --- | --- | --- |
| *s__Lactobacillus_kefiranofaciens* | **1** | 0 | 0 | 0 | 0.001 |
| *s__metagenome_g__unclassified_f__Rhizobiales_Incertae_Sedis* | **0.973711** | 0.026289 | 0 | 0 | 0.001 |
| *s__unclassified_c__Gammaproteobacteria* | **0.974063** | 0 | 0 | 0.008646 | 0.001 |
| *s__unclassified_d__Bacteria* | **0.935788** | 0.026616 | 0.031607 | 0.004991 | 0.001 |
| *s__unclassified_f__Erysipelotrichaceae* | **0.960422** | 0.006596 | 0 | 0 | 0.001 |
| *s__unclassified_f__Rhodobacteraceae* | **0.98804** | 0.000249 | 0.003987 | 0.001246 | 0.001 |
| *s__unclassified_f__Ruminococcaceae* | **0.984502** | 0.004428 | 0 | 0.004428 | 0.001 |
| *s__unclassified_g__Bosea* | **1** | 0 | 0 | 0 | 0.001 |
| *s__uncultured_bacterium_g__Paludicola* | **0.94586** | 0.035828 | 0.000265 | 0.003185 | 0.001 |
| *s__uncultured_bacterium_g__Pseudorhodobacter* | **0.974014** | 2.77623542476402e-05 | 0.025153 | 0.000222 | 0.001 |
| *s__Christensenella_minuta* | 0.001127 | **0.987323** | 0 | 0.002535 | 0.001 |
| *s__Gordonibacter_massiliensis* | 0 | **0.963345** | 0 | 0.030546 | 0.001 |
| *s__Robinsoniella_peoriensis* | 0 | **0.970446** | 8.65665956820582e-05 | 0.029294 | 0.001 |
| *s__Tsukubamonas_globosa* | 0.067078 | **0.930546** | 3.18681568823837e-05 | 0.001901 | 0.001 |
| *s__unclassified_f__Christensenellaceae* | 0 | **0.941176** | 0 | 0.009804 | 0.001 |
| *s__unclassified_f__Eggerthellaceae* | 0 | **1** | 0 | 0 | 0.001 |
| *s__unclassified_f__Lachnospiraceae* | 0.001335 | **0.990689** | 0.002102 | 0.002102 | 0.001 |
| *s__unclassified_f__Oscillospiraceae* | 0 | **0.962761** | 0 | 0.024826 | 0.001 |
| *s__unclassified_g__Acetobacterium* | 0 | **1** | 0 | 0 | 0.001 |
| *s__unclassified_g__Anaerotruncus* | 0 | **0.940959** | 0 | 0.02952 | 0.001 |
| *s__unclassified_g__Faecalitalea* | 0 | **1** | 0 | 0 | 0.001 |
| *s__unclassified_g__Lachnoclostridium* | 0 | **0.940583** | 0 | 0.049514 | 0.001 |
| *s__uncultured_bacterium_g__Acetobacterium* | 0 | **0.967456** | 0.000247 | 0.02071 | 0.002 |
| *s__uncultured_bacterium_g__Anaerovorax* | 0.000909 | **0.939034** | 5.11456628477905e-05 | 0.058613 | 0.001 |
| *s__uncultured_bacterium_g__Butyricicoccus* | 0 | **0.910606** | 0.00101 | 0.055556 | 0.001 |
| *s__uncultured_bacterium_g__unclassified_o__Clostridia_UCG_014* | 0 | **1** | 0 | 0 | 0.001 |
| *s__Mycobacterium_tuberculosis_g__Mycobacterium* | 0 | 4.52591083955646e-05 | **0.987508** | 0.010183 | 0.002 |
| *s__Phaseolus_vulgaris_g__unclassified_o__Chloroplast* | 0 | 0 | **0.960784** | 0.013072 | 0.001 |
| *s__Pseudomonas_syringae_pv__actinidiae* | 0 | 0 | **0.956938** | 0.021531 | 0.001 |
| *s__Streptococcus_cristatus* | 0 | 0 | **0.982143** | 0.005952 | 0.001 |
| *s__unclassified_g__Aquabacterium* | 0.000413 | 0 | **0.996279** | 0 | 0.001 |
| *s__unclassified_g__Methylophilus* | 1.23937910205435e-05 | 0 | **0.994695** | 0.00525 | 0.001 |
| *s__unclassified_g__Nevskia* | 0 | 0 | **0.988411** | 0.009657 | 0.001 |
| *s__uncultured_Cytophagales_bacterium_g__unclassified_f__NS11_12_marine_group* | 0.000296 | 0 | **0.993351** | 0.000665 | 0.001 |
| *s__uncultured_Legionella_sp__g__Legionella* | 0 | 0.003717 | **0.929368** | 0.02974 | 0.001 |
| *s__uncultured_Verrucomicrobia_bacterium_g__unclassified_f__UBA12409* | 0 | 0 | **0.999258** | 0.000371 | 0.001 |
| *s__uncultured_bacterium_g__Arcicella* | 0 | 0 | **0.994885** | 0.000853 | 0.001 |
| *s__uncultured_bacterium_g__Microvirga* | 0 | 0 | **0.979592** | 0.003401 | 0.001 |
| *s__uncultured_bacterium_g__Parasediminibacterium* | 0.003065 | 0 | **0.972414** | 0 | 0.001 |
| *s__uncultured_bacterium_g__Undibacterium* | 0 | 0 | **0.990099** | 0.00165 | 0.001 |
| *s__uncultured_bacterium_g__unclassified_f__Beijerinckiaceae* | 0 | 0 | 0.034014 | **0.907959** | 0.001 |

Note: Green indicates potentially beneficial microbes; red indicates potentially harmful microbes; purple indicates unclear (beneficial vs. harmful).yellow indicates indicator taxa for the C group; blue indicates indicator taxa for the G group; dark green indicates indicator taxa for the Z group; brown indicates indicator taxa for the ZG group.
